# The Proteome of Antibody-Mediated Rejection: From Glomerulitis to Transplant Glomerulopathy

**DOI:** 10.3390/biomedicines10030569

**Published:** 2022-02-28

**Authors:** Bertrand Chauveau, Anne-Aurélie Raymond, Sylvaine Di Tommaso, Jonathan Visentin, Agathe Vermorel, Nathalie Dugot-Senant, Cyril Dourthe, Jean-William Dupuy, Julie Déchanet-Merville, Jean-Paul Duong Van Huyen, Marion Rabant, Lionel Couzi, Frédéric Saltel, Pierre Merville

**Affiliations:** 1 CHU de Bordeaux, Service de Pathologie, Hôpital Pellegrin, Place Amélie Raba Léon, F-33000 Bordeaux, France; 2 ImmunoConcEpT, CNRS, Université Bordeaux, UMR 5164, 146 Rue Léo Saignat, F-33000 Bordeaux, France; jonathan.visentin@duke.edu (J.V.); julie.dechanet-merville@u-bordeaux.fr (J.D.-M.); lionel.couzi@chu-bordeaux.fr (L.C.); pierre.merville@chu-bordeaux.fr (P.M.); 3 Plateforme Oncoprot, TBM-Core US 005, Université Bordeaux, F-33000 Bordeaux, France; anne-aurelie.raymond@inserm.fr (A.-A.R.); sylvaine.di-tommaso@inserm.fr (S.D.T.); cyril.dourthe@inserm.fr (C.D.); frederic.saltel@inserm.fr (F.S.); 4 INSERM UMR1312, BoRdeaux Institute of onCology (BRIC), Université Bordeaux, F-33000 Bordeaux, France; 5 Laboratoire d’Immunologie et Immunogénétique, CHU de Bordeaux, Hôpital Pellegrin, Place Amélie Raba Léon, F-33000 Bordeaux, France; 6 CHU de Bordeaux, Service de Néphrologie, Transplantation Dialyse, Aphérèses, Hôpital Pellegrin, Place Amélie Raba Léon, F-33000 Bordeaux, France; agathe.vermorel@chu-bordeaux.fr; 7 Plateforme d’Histopathologie, TBM-Core US 005, Université Bordeaux, F-33000 Bordeaux, France; nathalie.senant@inserm.fr; 8 Plateforme Protéome, Université Bordeaux, F-33000 Bordeaux, France; jean-william.dupuy@u-bordeaux.fr; 9 Assistance Publique—Hôpitaux de Paris (AP-HP), Service de Pathologie, Hôpital Necker, F-75015 Paris, France; jean-paul.duong@aphp.fr (J.-P.D.V.H.); marion.rabant@aphp.fr (M.R.); 10 INSERM U1151, F-75730 Paris, France

**Keywords:** antibody-mediated rejection, glomerulus, proteomics, transplant glomerulopathy, kidney transplantation

## Abstract

Antibody-mediated rejection (ABMR) is the leading cause of allograft failure in kidney transplantation. Its histological hallmark is represented by lesions of glomerulitis i.e., inflammatory cells within glomeruli. Current therapies for ABMR fail to prevent chronic allograft damage i.e., transplant glomerulopathy, leading to allograft loss. We used laser microdissection of glomeruli from formalin-fixed allograft biopsies combined with mass spectrometry-based proteomics to describe the proteome modification of 11 active and 10 chronic active ABMR cases compared to 8 stable graft controls. Of 1335 detected proteins, 77 were deregulated in glomerulitis compared to stable grafts, particularly involved in cellular stress mediated by interferons type I and II, leukocyte activation and microcirculation remodeling. Three proteins extracted from this protein profile, TYMP, WARS1 and GBP1, showed a consistent overexpression by immunohistochemistry in glomerular endothelial cells that may represent relevant markers of endothelial stress during active ABMR. In transplant glomerulopathy, 137 proteins were deregulated, which favor a complement-mediated mechanism, wound healing processes through coagulation activation and ultimately a remodeling of the glomerular extracellular matrix, as observed by light microscopy. This study brings novel information on glomerular proteomics of ABMR in kidney transplantation, and highlights potential targets of diagnostic and therapeutic interest.

## 1. Introduction

Short-term allograft survival has significantly increased over past decades in kidney transplantation thanks to improvements in immunosuppressive strategies. In contrast, long-term allograft survival did not increase proportionately and has become a major issue [1]. Currently, antibody-mediated rejection (ABMR) is considered as the leading cause of kidney allograft failure, involved in about two-thirds of cases [2]. Antibody-mediated rejection is primarily an endothelial disease, mediated by donor-specific antibodies (DSA) that target human leukocyte antigens (HLA) or non-HLA antigens. Bound DSA to endothelial cells lead to recruitment of inflammatory cells and injuries (from activation to cell lysis), which can be detected in an allograft biopsy by lesions of microvascular inflammation: glomerulitis and peritubular capillaritis. These mechanisms are thought to be complement-mediated or not, the latter in up to 50% of cases [3]. The identification of complement mediation is based on the histological deposition of the complement fragment C4d on the peritubular capillaries, that can be detected by immunohistochemistry or immunofluorescence [4]. These histological lesions (microvascular inflammation and C4d), as well as the detection of DSA in the serum of patients, are currently the hallmark criteria of active ABMR definition, according to the 2019 Banff international classification [5]. The diagnosis can be retained even if not all criteria are present, as proposed surrogate markers allow several combinations to be accepted (e.g., C4d negative ABMR may be diagnosed when a significant microvascular inflammation is present in addition to the detection of DSA).

The Banff classification recognizes chronic ABMR if at least one of the following chronic tissue injuries is present: double contours of the glomerular basement membrane (called transplant glomerulopathy), severe multilayering of the peritubular capillary basement membrane or arterial intimal fibrosis of new onset without any other cause [6]. Concomitant active and chronic microvascular histological lesions, such as glomerulitis and transplant glomerulopathy in the same allograft biopsy, are a common finding and are defined as chronic active ABMR. The observed multilayering of basement membranes, assessed in the first place by ultrastructural analysis, is considered to be induced by repeated, prolonged and/or sublytic endothelial damages, that are known to promote proinflammatory, procoagulant and proliferative-restorative changes of the endothelial cells and their environment [7]. While transplant glomerulopathy is not uncommon, with a cumulative incidence estimated of approximately 20% at 5 years of transplantation, and is associated with proteinuria and declining allograft function [8,9], the literature lacks an in-depth exploration of the deregulated proteins observed in this severe entity [10,11]. Changes in the extracellular matrix have been recently described by proteomics during active ABMR [12]. However, to our knowledge, transplant glomerulopathy has not been extensively described.

The understanding of the pathophysiological mechanisms of active ABMR has greatly improved with transcriptomic approaches from frozen samples [13,14], which notably highlighted the major involvement of macrophages and NK cells, interferon gamma and activated endothelial cells during active ABMR, and revealed its C4d negative phenotype [4]. However, the molecular mechanisms involved in antibody-mediated processes (chronic or not) and induced tissue modifications are still incompletely elucidated. Indeed, active ABMR is quite responsive to therapies such as intravenous immunoglobulins or plasmapheresis on a short-term perspective. Yet, it still represents a turning point responsible for a severe reduction in the lifespan of the graft on a middle and long-term perspective, as current therapeutic strategies fail to prevent the genesis of chronic tissue injuries [15,16]. Personalized treatment for active ABMR is thus undergoing extensive research but remains to date an unmet need. Likewise, the responsiveness of chronic active ABMR to current therapies is disappointing, possibly due to already advanced and irreversible chronic allograft damage. Two recent low-scale studies showed potential benefits of anti-interleukin-6 therapies in transplant glomerulopathy [17,18], but this needs to be validated in large controlled clinical trials. A better characterization of the effector mechanisms of active ABMR and transplant glomerulopathy is needed to potentially reveal new therapeutic targets and thus improve the immune component of long-term allograft survival.

An increasing interest in high-throughput proteomic analysis is emerging in the field of renal diseases, especially in the glomerular diseases [19]. Proteomics allow a holistic view of tissue proteins, by analyzing not only the intracellular but also the extracellular compartment, that is less explored by transcriptomic approaches [20]. Proteomics also provides a better correlation with in situ protein expression technics such as immunohistochemistry than transcriptomics. Combining mass spectrometry-based proteomics with laser microdissection allows relevant analyzes of specific tissue compartment such as glomeruli from small amounts of tissue. Glomerular proteome characterization notably revealed DNAJB9 as an immunohistochemical diagnosis biomarker of fibrillary glomerulonephritis [21,22], and allowed the discovery of several potential target antigens for autoantibodies in membranous nephropathy [23,24,25,26]. To our knowledge, a comprehensive characterization of the ABMR proteome in both its active and chronic forms has never been performed.

Herein, we used glomerular microdissection coupled with mass spectrometry-based proteomics from formalin-fixed and paraffin embedded (FFPE) kidney biopsy samples. Our goals were to (1) describe the protein profile of glomerular injuries in active antibody-mediated rejection i.e., glomerulitis in human kidney transplantation, (2) highlight potentially relevant markers of diagnostic interest for pathologists and (3) characterize the proteome of transplant glomerulopathy with emphasis on the extracellular matrix.

## 2. Materials and Methods

### 2.1. Selection of the Cohort

**Figure 1:**
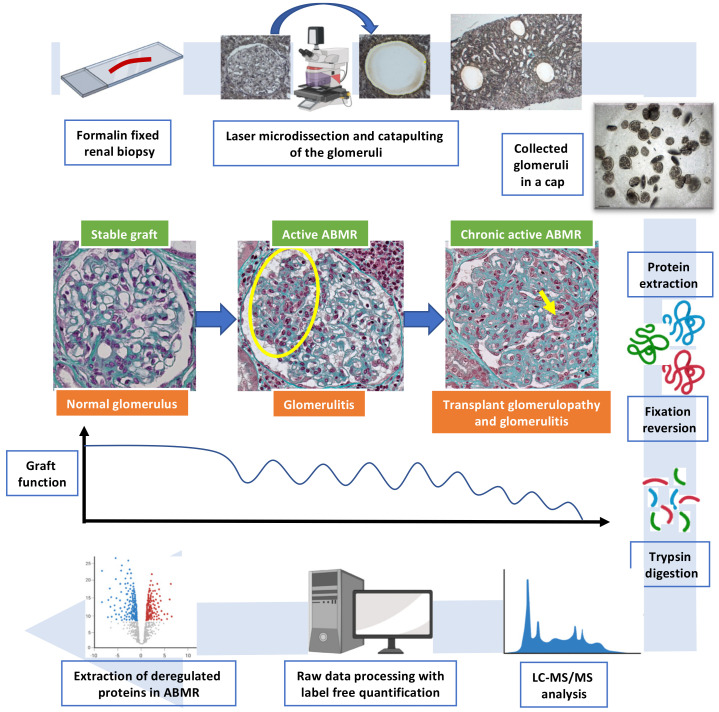
Overall analytical strategy of the study. The histological hallmark of kidney active antibody-mediated rejection is glomerulitis i.e., inflammatory cells within the glomerular capillary loops. This lesion can be seen by light microscopy (yellow circle and arrow, Masson’s trichrome staining, original magnification ×400). Prolonged injuries lead to an expansion of the extracellular matrix (stained in green with Masson’s trichrome) and a duplication of the glomerular basement membrane called transplant glomerulopathy. These lesions are associated with graft dysfunction, proteinuria and ultimately allograft loss. Herein we performed a bottom-up proteomic approach on laser microdissected glomeruli during active ABMR, chronic active ABMR and stable graft patients from formalin-fixed and paraffin-embedded allograft biopsies. The key technical steps are presented by following the blue arrow. Abbreviations: ABMR, antibody-mediated rejection.

This is a descriptive, retrospective single-center study analyzing kidney allograft biopsies in active ABMR, chronic active ABMR and in stable immunological state. The overall analytical strategy is described in Figure 1.

All included cases consisted of renal allograft biopsies already performed for diagnosis purposes from April 2011 to September 2018 at the Bordeaux University Hospital. The cohort consisted of three groups: 11 patients with active ABMR (aABMR), 10 with chronic active ABMR (caABMR) and 8 stable graft controls (SG). The diagnoses of aABMR and caABMR were in accordance with the 2017 Banff classification, and all patients had anti-HLA DSA in their serum at the time of the diagnosis of rejection. As we carefully focused our analysis on the glomeruli, all selected biopsies had histological glomerulitis (Banff g score greater than or equal to 1, g ≥ 1). Chronic ABMR was defined by light microscopy (≥cg1b). Biopsies with mixed rejection, displaying both antibody-mediated and T-cell mediated rejection, were not included, but borderline infiltrate was admitted. Biopsies included in the SG group consisted of one-year protocol biopsies in patients with an estimated Glomerular Filtration Rate (eGFR) greater than 40 mL/min/1.73 m^2^, no clinical proteinuria (below 50 mg/mmol), no anti-HLA DSA and no history of rejection or BK virus infection. These samples had no acute injury nor chronic glomerular lesion (cg0 mm0). For all cases, immunofluorescence study with antibodies targeting IgA, IgG, IgM, C3, Kappa and Lambda was negative. C4d status was assessed by immunohistochemistry. As required by the local institutional ethics board, patients for whom kidney biopsy was eligible were contacted and had the legal time to express their opposition. Our clinical database had a French CNIL (Commission Nationale Informatique et Libertés) final agreement, decision 2009-413, n° 1357154, 2 July 2009.

### 2.2. Concise Proteomic Method

For each case, fifty non-sclerotic glomerular sections were microdissected from 5 μm-thick FFPE tissue section with a PALM type 4 laser microdissector (Zeiss, Oberkochen, Germany). Two replicates were performed for each biopsy. Proteins were extracted with formalin cross-link reversion, digested by trypsin and proteolytic peptides were subjected to mass-spectrometry (MS) analysis, as previously described [27,28,29]. Briefly, the Proteome Discoverer 1.4 Software (Thermo FisherScientific, Illkirch, France) used the Mascot 2.5 algorithm to identify proteins (*Homo sapiens* database, 73,658 entries, Reference Proteome Set, release date: 13 December 2018 from http://www.uniprot.org/ website (accessed on 25 April 2019). Two missed enzyme cleavages were allowed. Methionine oxidation, lysine acetylation, asparagine and glutamine deamidations were selected as dynamic modifications, and cysteine carbamidomethylation as static modification. Peptides were validated with Proline software [30]. Only peptides with 1.0% false-discovery rate (FDR), calculated using the Mascot “decoy” option, and a pretty rank = 1 were retained. Proteins were identified with ≥2 specific peptides and FDR < 1.0%. Label-free quantification of MS1 level by extracted ion chromatograms (XIC) was carried out with parameters indicated previously [27]. The mass spectrometry proteomics data have been deposited to the ProteomeXchange Consortium via the PRIDE [31] partner repository with the dataset identifier PXD021852. Detailed proteomic methods are provided in Supplemental Materials.

### 2.3. Immunohistochemical Analysis

Immunohistochemical analyses were performed as previously described [28] and specific details are provided in Supplemental Materials. All staining procedures were performed using an automated autostainer (Dako-Agilent, Santa Clara, CA, USA). Five commercial primary antibodies were used, targeting thymidine phosphorylase (TYMP), tryptophan—tRNA ligase, cytoplasmic (WARS1), coronin-1A (CORO1A), guanylate-binding protein 1 (GBP1) and EF-hand domain-containing protein D2 (EFHD2).

Histological glomerular scores evaluating global intensity of staining were assessed for each antibody. For WARS1, GBP1 and TYMP antibodies, scores were calculated for each case by assessing the mean intensity of staining of the glomeruli, by visually evaluating the number of positive glomeruli and their respective intensity of staining (0 to 3+). Only cases with a minimum of 4 non-sclerotic glomeruli were considered for this semi-quantitative evaluation, where most cases had a diffuse pattern of staining (i.e., a majority of glomeruli showed positivity). For the CORO1A and EFHD2 antibodies, scores were assessed by calculating the mean number of positive cells per glomerular section with a visual enumeration. Only cases with at least 8 non-sclerotic glomeruli were assessed for this quantitative enumeration, as glomerulitis is often a focal lesion.

C4d positivity on peritubular capillaries was semi-quantitatively scored according to the Banff guidelines. A score 0 was considered negative, and positive otherwise (score 1 to 3). No Banff rule exist for the scoring of C4d positivity in the glomeruli. As such a semi-quantitative score from 0 to 3 was assessed for each case by visually evaluating the mean intensity of glomerular staining.

### 2.4. Bioinformatical Analysis of Proteomics Data and Statistics

Mann-Whitney U tests were used to compare protein abundance distribution between the three groups. For each duplicate, only the mean protein abundance was considered. Coefficients of variation were calculated for each protein and duplicates as the standard deviation (SD) divided by the mean. For each comparison, only the proteins with a fold-change above 1.5 (overrepresentation) or lesser than 0.66 (underrepresentation) were retained as deregulated. *p*-values were further corrected using the Benjamini-Hochberg multiple test correction algorithm [32], and the threshold of statistical significance was considered at 0.05. Keratins from the epidermis (contaminants) were manually removed. To reveal the main biological processes of the protein sets, overrepresentation analyzes were performed using the enrichGO function of the R package clusterProfiler, version 4.0.5 [33], with a distinct analysis of overrepresented and underrepresented proteins. We used the 1335 detected proteins of our study as background. Biological processes were in accordance with the Gene Ontology terminology, and the threshold of statistical significance was considered at 0.05 for adjusted *p*-values. Benjamini-Hochberg correction was performed for each comparison considering every biological process involving at least 3 proteins. Redundant biological processes were manually removed.

To study the extracellular matrix proteome modifications, we only considered proteins that were recognized by the Human matrisome database from the Matrisome Project [34], using the Matrisome Annotator tool. Distributions of each protein were compared by performing non-parametric Mann-Whitney U tests. *p*-values were corrected using the Benjamini-Hochberg multiple test correction algorithm.

All statistical analyses were performed using the R software, version 4.1.1 [35]. Plots were performed using the ggplot2 package, version 3.3.5, and correlation analyzes with the cor.test function.

## 3. Results

### 3.1. Demographical Characteristics of the Cohort and Analytical Strategy

We initially included 34 patients. Five patients were secondarily excluded: 3 due to lack of remaining material in the paraffin block, one due to a positive BK viremia at the time of biopsy and one because of unusable spectrometric data. Overall, there were 8 patients in the SG group and 21 patients with ABMR features, including 11 in the aABMR group and 10 in the caABMR group. Supplementary Figure S1 describes the fully detailed flow-chart of the study. The ABMR groups mainly consisted of male patients, 8/11 and 7/10 for the aABMR and caABMR group, respectively, with a deceased donor (8/11 in the aABMR group, 10/10 in the caABMR group). All patients had anti-HLA DSA, with a predominance of pre-existing DSA (i.e., detected prior transplantation) for the aABMR group (7/11) while all patients in the caABMR group had de novo DSA. Patients in the caABMR tended to have a lower kidney allograft function and heavier proteinuria. They also all showed glomerulitis (g > 0) as well as transplant glomerulopathy (cg > 0). More patients were C4d positive on the peritubular capillaries in the caABMR cases (5/10) than in the aABMR cases (2/11). The control stable graft group was predominantly male (5/8), all with a satisfactory allograft function (eGFR above 40 mL/min/1.73 m^2^) and with no significant proteinuria. They consisted of protocol biopsies with no evidence of rejection, either histologically (no glomerulitis, no C4d deposits) or biologically (no DSA in serum). Table 1 summarizes all relevant clinical, biological and histological characteristics. Figure 1 describes the overall analytical strategy.

### 3.2. Proteomic Profiling of Acute Antibody-Mediated Glomerular Injuries i.e., Glomerulitis

**Figure 2:**
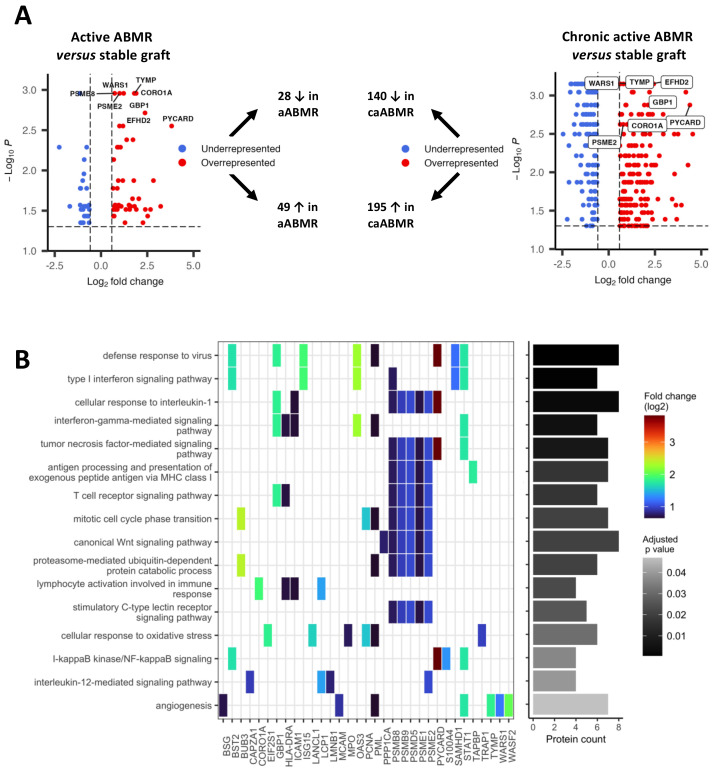
Characterization of the proteome of glomerulitis, with volcano plots showing significantly deregulated proteins in the two ABMR/SG comparisons (**A**) and a heatmap-like plot displaying the main enriched biological processes (**B**). (**A**) Protein abundances were compared using Mann-Whitney U tests with a Benjamini-Hochberg correction of the *p*-values. The significance threshold for adjusted *p*-values was set at 0.05. For clarity, only the deregulated proteins are showed in the volcano plots for each comparison. Overrepresentation was defined as a fold change above 1.5 and an underrepresentation when the fold change was lesser than 0.66. (**B**) Biological processes follow the Gene Ontology terminology, identified by performing overrepresentation analyzes using the enrichGO function of the clusterProfiler R package version 4.0.5. Only the biological processes involving overrepresented proteins of the aABMR/SG comparison are presented here. Fold changes (log2) are displayed with a color gradient. Number of proteins enriched in each biological process is presented as a bar plot on the right-hand side. Redundant biological processes were manually removed. Abbreviations: aABMR, active antibody-mediated rejection; caABMR, chronic active antibody-mediated rejection; SG, stable graft control.

A total of 1335 proteins were detected and quantified for all samples, with a median coefficient of variation of 14.9% between duplicates. As shown in Figure 2A, 77 proteins were significantly deregulated in the aABMR group compared to the SG one (adjusted *p*-value < 0.05), considered to reflect changes occurring during glomerulitis. They consisted of 49 overrepresented (fold-change above 1.5) and 28 underrepresented (fold-change lesser than 0.66) proteins. Similarly, there were 335 proteins deregulated in the caABMR compared to the SG group, deemed to reflect changes occurring during both glomerulitis and transplant glomerulopathy. They consisted of 195 overrepresented and 140 underrepresented proteins. Table 2 shows the top-25 most significant proteins for each comparison and in accordance with adjusted *p*-values. Supplementary Tables S1 and S2 show the whole protein sets. Interestingly, 59 proteins were both deregulated in the aABMR/SG as well as in the caABMR/SG comparison, representing 77% (59/77) of the deregulated proteins of the aABMR/SG comparison. These proteins displayed a similar deregulation pattern in the two comparisons (Supplementary Figure S2), where no statistical difference between the two distributions of fold-change was seen (*p* = 0.58, Wilcoxon signed-rank test).

To describe biological processes enriched in glomerulitis, we performed overrepresentation analyzes, considering the aABMR/SG comparison We performed a distinct assessment between overrepresented and underrepresented proteins. Figure 2B shows the main Gene Ontology biological processes. The set of 49 overrepresented proteins mainly referred to immune-related pathways, primarily cytokine-mediated signaling pathways, and, overall, mimicking a defense to virus response (*p* = 5.6 × 10^−4^). The major cytokine-mediated processes included both type I and II interferons (*p* = 7.9 × 10^−4^ and *p* = 0.004, respectively), interleukin-1 (*p* = 0.002) and tumor necrosis factor (TNF, *p* = 0.007). Other relevant immune pathways included immunoproteasome for antigen processing (*p* = 0.017) and lymphocyte activation (T-cell receptor signaling pathway, *p* = 0.017). Lastly, a vascular process was also significant (angiogenesis, *p* = 0.047). Considering the underrepresented proteins, there were no significantly enriched pathway after the Benjamini-Hochberg correction of the *p*-values.

Glomerulitis is not a dichotomous histological lesion. According to the Banff classification, glomerulitis is semi-quantitatively assessed from 0 (no glomerulitis) to 3 (glomerulitis in more than 75% of glomeruli) and, as such, is deemed to reflect the degree of antibody-mediated injury. We therefore studied the Spearman’s correlation between protein abundance and the glomerulitis semi-quantitative g score among the proteins deregulated in glomerulitis. This allowed us to characterize proteins whose abundance best match with the g score and could therefore reflect antibody-mediated damage. CORO1A (rho = 0.92, *p* = 1.2 × 10^−9^), LCP1 (rho = 0.91, *p* = 5.6 × 10^−9^) and EFHD2 (rho = 0.86, *p* = 7.3 × 10^−7^) were the most correlated proteins with the glomerulitis score (Supplementary Figure S3). Table 3 shows the top-10 ranked proteins with a positive correlation with glomerulitis. Interestingly, CR1, a well-known complement inhibitor protein, was negatively correlated with the glomerulitis score (rho = −0.79, *p* = 2.8 × 10^−7^).

### 3.3. Immunohistochemical Validation of 5 Proteins Overrepresented in Glomerulitis

To highlight diagnostic markers of ABMR for pathologists, we selected 5 proteins significantly overrepresented in both active ABMR groups for immunohistochemical validation. The selection method was based on the most significant proteins overrepresented in the aABMR/SG comparison and on the proteins most correlated with the glomerulitis score. We retained TYMP, GBP1, WARS1, CORO1A and EFHD2. Immunohistochemistry was carried out for each case with sufficient remaining material.

**Figure 3:**
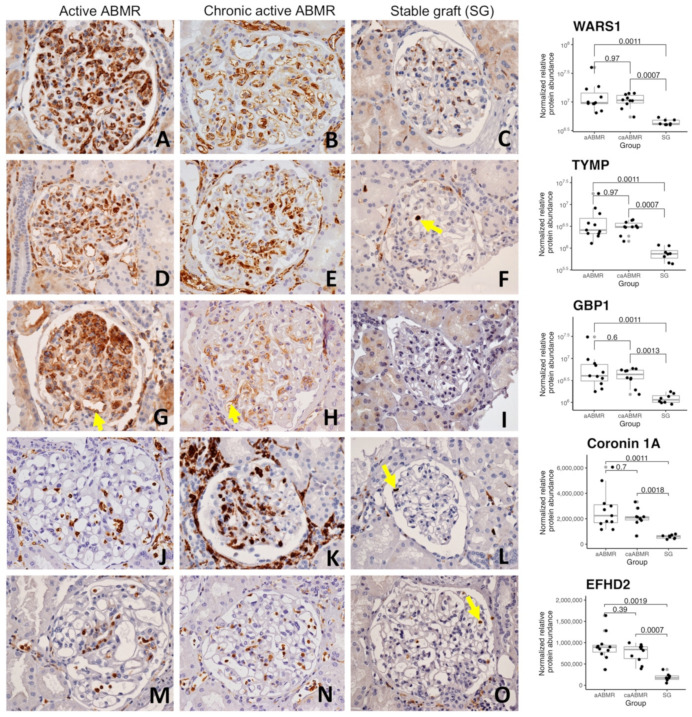
Highlighting glomerulitis with illustrative cases of glomerular immunostains for WARS1, TYMP, GBP1, CORO1A and EFHD2 in active (left), chronic active antibody-mediated rejection (middle) and stable graft control cases (right), original magnification × 400 ((**A**)–(**O**), respectively). Corresponding proteomic results depending on groups are displayed on the right-hand side, with adjusted *p*-values for each comparison (Mann-Whitney U tests and Benjamini-Hochberg correction). (**A**,**B**) show a diffuse cytoplasmic overexpression of WARS1 on endothelial cells in a glomerulus of an active and chronic active ABMR cases, with a strong and moderate positivity, respectively, compared to the constitutive weak and segmental endothelial staining observed in the stable graft control (**C**). (**D**,**E**) show a diffuse and moderate to strong overexpression of TYMP in endothelial cells, but also a strong staining in endocapillary inflammatory cells (**E**). (**F**) Rare inflammatory cells are strongly stained for TYMP (arrow), without endothelial staining in the glomerulus. (**G**) shows a strong staining for GBP1 in endothelial (arrow) and inflammatory cells in the glomeruli (focus of glomerulitis at the top). (**H**) displays a segmental and weak to moderate staining in some endothelial (arrow) and inflammatory cells. (**I**) No specific staining for GBP1 in a stable graft case. (**J**,**K**) show a strong, nuclear and cytoplasmic staining for CORO1A in noticeably all inflammatory cells, wherever they are. Many positive cells are visible in the glomerulus of both ABMR cases, with a segmental and global lesion of glomerulitis, respectively. (**L**) shows the same expression pattern of CORO1A, but limited to a few inflammatory cells in the peritubular capillaries or the interstitium, and just one cell in the glomerulus (arrow). (**M**,**N**) show a moderate to strong, nuclear and cytoplasmic staining for EFHD2 antibody in some inflammatory cells in the glomeruli of both ABMR cases. Of note, stained inflammatory cells are also seen in the nearby peritubular capillaries. (**O**) shows a similar pattern of expression of EFHD2, but with globally much fewer positive cells, limited in the glomerulus to 2 moderately stained neutrophils (arrow). Abbreviations: ABMR, antibody-mediated rejection.

Figure 3 shows illustrative cases of aABMR, caABMR and SG for each antibody. All five antibodies showed a cytoplasmic and, to some extent, a nuclear positivity. They all stained inflammatory infiltrates, but with various strength and pattern (diffuse, focal or scattered cells). CORO1A and EFHD2 almost exclusively stained inflammatory cells, almost all for CORO1A, much less for EFHD2 (scattered pattern). A diffuse inflammatory cell staining was observed with TYMP, while a focal and scattered positivity was seen with WARS1 and GBP1. Interestingly WARS1, TYMP and GBP1 also stained endothelial cells in the ABMR cases, both in the glomeruli and peritubular capillaries, even without prominent inflammatory cells. WARS1 and TYMP mostly showed a global and diffuse positivity in endothelial cells in the glomeruli while GBP1 displayed a focal (not every glomerulus) and segmental (not the entire glomerulus) positivity. There were no overt differences in the staining pattern of these 5 antibodies between the active and chronic active ABMR cases. Overall, the staining pattern observed for each antibody supported the morphological and functional analyzes described earlier: EFHD2 and CORO1A, whose proteomic abundance was highly correlated with glomerulitis, stained inflammatory cells in the glomeruli while WARS1, TYMP and GBP1, proteins that are related to the interferon environment, also highlighted endothelial cells during ABMR.

To evaluate the potential of these 5 proteins as immunomarkers of ABMR, we first studied the correlation between proteomic abundance and immunohistochemical glomerular expression. For each slide and each antibody, a pathologist (BC) visually assessed a semi-quantitative histological glomerular score of the in situ expression. For CORO1A and EFHD2, the mean number of positive inflammatory cells was calculated because CORO1A and EFHD2 only stain inflammatory infiltrate. Conversely, for TYMP, WARS1 and GBP1, a semi-quantitative score per glomerular section was assessed and averaged, as they all stain both inflammatory and endothelial cells (see also Section 2). We observed a significant correlation between the histological glomerular scores and the spectrometric protein abundances for all antibodies, with a Spearman’s correlation coefficient rho = 0.80 (*p* = 1.6 × 10^−5^) for WARS1 (n = 21 analyzed cases), rho = 0.90 (*p* = 3.7 × 10^−6^) for TYMP (n = 22) and rho = 0.93 (*p* = 1.9 × 10^−8^) for GBP1 (n = 18). Spearman’s correlation coefficient was of 0.82 (*p* = 2.6 × 10^−4^) for CORO1A (n = 15) and rho = 0.70 (*p* = 5.8 × 10^−3^) for EFHD2 (n = 15). Supplementary Table S3 provides the detailed scores for each case.

Since glomerulitis is a lesion seen in active ABMR, either acute or chronic active, a reliable immunomarker of activity should be independent of chronicity. Here, we did not observe a morphological or quantitative difference for these 5 proteins between acute and chronic active cases of ABMR (Figure 3). Similarly, as active ABMR can also be C4d negative, i.e., without C4d deposits, we compared the proteomic abundances of these 5 proteins between C4d positive and C4d negative cases. We did not observe any statistical difference (Supplementary Figure S4).

### 3.4. Proteomic Profiling of Chronic Antibody-Mediated Glomerular Injuries i.e., Transplant Glomerulopathy

We compared the caABMR to the aABMR group, considered to reflect the changes occurring during antibody-mediated transplant glomerulopathy. Here, 137 proteins were deregulated, including 99 overrepresented and 38 underrepresented proteins. In accordance with adjusted *p*-values, the top-25 most significant proteins are presented in Table 4 and the whole protein set is listed in Supplementary Table S4.

**Figure 4:**
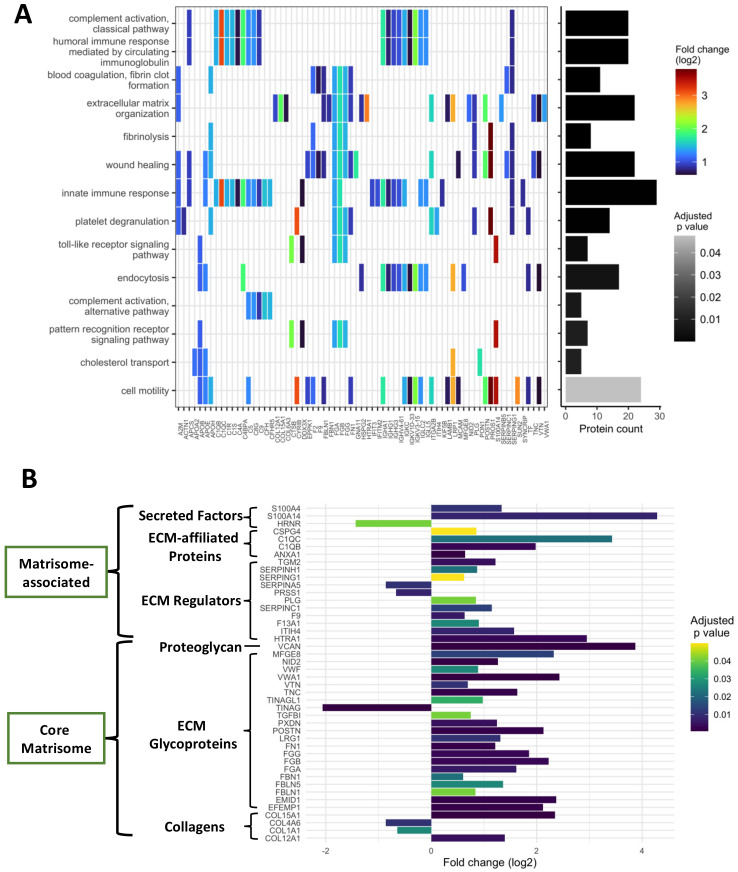
Characterization of the proteome of transplant glomerulopathy, with a heatmap-like plot displaying the main enriched biological processes (**A**) and a description of the extracellular matrix remodeling (**B**). (**A**) Biological processes follow the Gene Ontology terminology, identified by performing overrepresentation analyzes, using the enrichGO function of the clusterProfiler R package version 4.0.5. Only the biological processes involving overrepresented proteins of the caABMR/aABMR comparison are presented here. Fold changes (log2) are displayed with a color gradient. Number of proteins enriched in each biological process is presented as a bar plot on the right hand side. Redundant biological processes were manually removed. (**B**) Extracellular matrix proteins (ECM) differentially represented in transplant glomerulopathy. Fold changes are illustrated as a bar plot. A grey-scale gradient reflects the *p*-values. Each protein is annotated by its ECM category according to the Matrisome database. Abbreviations: aABMR, active antibody-mediated rejection; caABMR, chronic active antibody-mediated rejection; SG, stable graft control; ECM, extracellular matrix.

As with glomerulitis, we examined the biological processes enriched in transplant glomerulopathy by performing overrepresentation analyzes, with a distinct assessment of overrepresented and underrepresented proteins. The 99 overrepresented protein set mainly referred to B-cell mediated immunity (*p* = 1.67 × 10^−11^), complement activation and its regulation, with an emphasis on the classical pathway (*p* = 8.8 × 10^−14^) in comparison to the alternative pathway (*p* = 0.007). Other relevant pathways included blood coagulation (*p* = 1.4 × 10^−8^) and platelet activation (*p* = 1.2 × 10^−4^), wound healing (*p* = 2.9 × 10^−5^), extracellular matrix organization processes (*p* = 2.6 × 10^−6^) and phagocytosis (*p* = 0.004). Figure 4A summarizes the main Gene Ontology biological processes enriched. As for the underrepresented proteins, they referred to small molecule catabolic processes (*p* = 4.4 × 10^−7^), cellular response to xenobiotic stimulus (*p* = 0.002), lipid oxidation (*p* = 0.03), glutathione metabolic process (*p* = 0.04) and mitochondrial transmembrane transport (*p* = 0.04).

As with glomerulitis, transplant glomerulopathy is not a dichotomous histological lesion, but a progressive remodeling of the glomeruli in response to chronic antibody-mediated injuries. Two main findings are seen by light microscopy: duplication of the glomerular basement membrane and expansion of the mesangial matrix, each lesion being scored from 0 to 3 according to Banff scheme (cg and mm, respectively). Based on the 137 protein dataset of transplant glomerulopathy, we performed correlation analyzes with the sum of the two Banff chronic lesions of the glomeruli i.e., cg + mm. The top 10 ranked proteins are shown in Table 5. The most positively correlated proteins were CFH (rho = 0.90, *p* = 4.5 × 10^−5^), COL15A1 (rho = 0.88, *p* = 7.9 × 10^−5^) and POSTN (rho = 0.82, *p* = 2.6 × 10^−3^). Supplementary Figure S5 shows the plots of the correlation for the first three proteins of each analysis.

Extracellular matrix (ECM) remodeling is of paramount importance in the progression of renal diseases [10]. To our knowledge, there is no in-depth proteome characterization of transplant glomerulopathy in the literature. In the present study, out of the 1335 proteins that were quantified, 135 proteins were directly related to the ECM, matching with the Matrisome Project database. They were all categorized and sub-categorized according to the Matrisome Annotator tool (core matrisome or matrisome-associated proteins, collagen, glycoproteins, ECM-regulators…). Supplementary Table S5 shows the whole set of ECM proteins. Considering transplant glomerulopathy, 44 proteins were significantly deregulated in caABMR compared to SG cases, including 26 core matrisome proteins (4 collagens, one proteoglycan and glycoproteins for the rest of them) and 18 ECM-associated proteins (mainly categorized as ECM-regulators). Six proteins were underrepresented and 38 overrepresented in transplant glomerulopathy (Figure 4B). A recent ECM-related proteomics study in early active ABMR showed a decrease of some ECM proteins such as collagens (COL4A1, COL4A4) and laminins (LAMA5, LAMB2) but also of two podocyte specific proteins: NPHS1 and PTPTRO [12]. Herein we did not find a decrease in the ECM proteins described by Clotet-Freixas et al. We detected 5 podocyte-specific proteins (NPHS1, NPHS2, PTPRO, PODXL and KIRREL1) in which two (NPSH1 and PODXL) were significantly decreased in transplant glomerulopathy and only PODXL in glomerulitis (Supplementary Table S6).

## 4. Discussion

**Figure 5:**
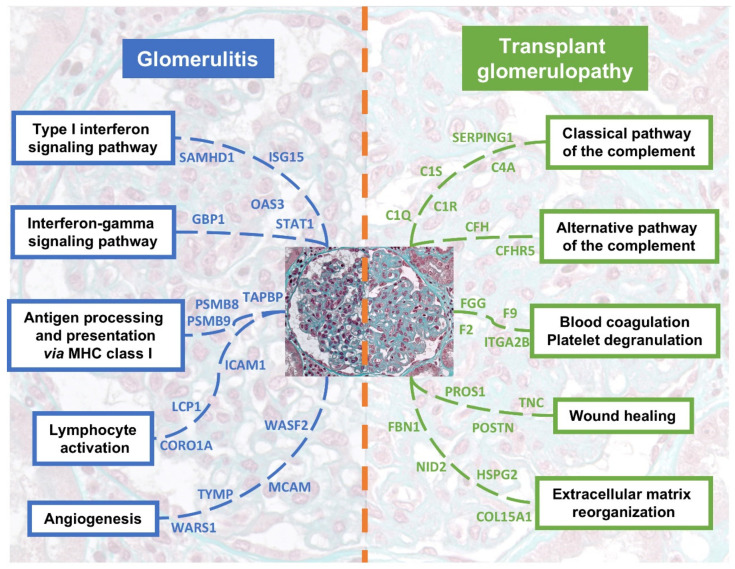
Integrative summary of the most relevant pathways enriched in glomerulitis and transplant glomerulopathy. Main upregulated proteins are depicted for each pathway. Please note that some proteins are shared between pathways, but we chose here to avoid any duplicate for clarity.

Antibody-mediated rejection is the leading cause of allograft failure in kidney transplantation. The efficacy of its treatment remains disappointing, particularly in the prevention of chronic tissue injury. Consequently, it is one of the major causes of the lack of improvement of long-term allograft survival. A better understanding of the underlying mechanisms occurring in this entity is an unmet need to expect potential therapeutic improvements. Herein we performed an in-depth proteomic characterization of ABMR in kidney transplantation at a glomerular scale. A visual and integrative summary of the main findings of this study is proposed in Figure 5. To our knowledge, this work represents the first study exploring both the active (glomerulitis) and chronic (transplant glomerulopathy) facets.

We described a protein profile of active antibody-mediated glomerular injuries i.e., glomerulitis, overall mimicking an antiviral stress response. Leukocyte activation was detected through many cytokine-mediated pathways, notably both type I and II (gamma) interferons, the latter having been particularly emphasized by microarrays at the RNA level [3,36]. Induced by these cytokines, our data also suggest a participation of the immunoproteasome (PSMB8 and PSMB9) for antigen processing and T-cell activation [37], a lymphocyte contingent known to be highly represented in antibody-mediated injuries [38]. The angiogenesis pathway likely represents an ongoing remodeling of the microcirculation in the glomeruli, as an indirect indicator of endothelial cell activation. This endothelial cell activation was particularly highlighted by our immunohistochemical findings with WARS1, TYMP and GBP1, all three displaying an overexpression in endothelial cells during ABMR. WARS1 is an IFNγ-induced protein, notably detected at the RNA level in active ABMR, whose some fragments have angiostatic effects and are therefore considered to play a role in vascular homeostasis [36,39,40]. As for TYMP, its effects are both pro-angiogenic and pro-thrombotic [41]. TYMP also enhanced C-X-C motif chemokine 10 (CXCL10) production in an in vitro model of rheumatoid arthritis [42], while this chemokine is also described as a urinary biomarker of ABMR [36,43]. GBP1 is also induced by IFNγ in notably both endothelial cells and monocytes. GBP1 globally inhibits cell proliferation and sensitivity to apoptosis and is crucial for the maturation of phagosomes for intracellular pathogens clearance [44,45].

With regards to ABMR diagnosis, the still unsolved issue of the direct detection of bound DSA in allograft biopsies led the Banff classification to evolving criteria of surrogate markers of endothelial injuries, such as C4d deposits and moderate microvascular inflammation (score g + ptc ≥ 2), or more recently molecular classifiers. In this context, highlighting an increased expression of interferon-related proteins in endothelial cells during aABMR may be of great interest for diagnostic purposes for pathologists. WARS1, TYMP and GBP1 could represent a concrete indicator of endothelial stress, as immunohistochemistry is still technically lighter and cheaper than molecular classification. The stability of their expression during chronic processes independently of the C4d status is another argument for their potential usefulness. Indeed up to 50% of active ABMR are C4d negative [3,4].

Considering chronic antibody-mediated glomerular injuries i.e., transplant glomerulopathy, our protein profile supports a complement-mediated injury process and endothelial activation that induce regional procoagulant changes and a healing process which finally lead to the extracellular matrix reorganization and expansion. These results are in accordance with the endothelial cells modifications described in chronic ABMR by Drachenberg et al. [7]. We confirmed the results of Nakorchevsky et al. [46], who explored the multifactorial lesions of interstitial fibrosis and tubular atrophy from whole biopsy through a proteogenomic approach, by underlining the importance of the complement system in the progression of chronic tissue injuries, here at a strict glomerular scale. Indeed our protein profile further revealed the major importance of both the classical and alternative pathways, but also the regulation of the complement system in chronic antibody-mediated lesions. Proximal components of the classical pathway (C1QB, C1QC, C1R, C1S) were notably overrepresented, consistent with an activation led by DSA bound to endothelial cell surface, but also were three terminal components (C5, C8G and C9). The regulation of the complement system was emphasized both in the classical (upregulated SERPING1 = C1 inhibitor and C4BPA) and in the alternative (CFH and CFHR5) pathways. These findings could support an established process of resistance to complement-induced injuries in transplant glomerulopathy. The complement factor H represented the leading protein in transplant glomerulopathy, and was highly correlated with the glomerular chronicity score cg + mm. After an initial trigger of the classical pathway through DSA binding on endothelial cells, CFH is thought to counteract the amplification of the C3 convertase genesis by the alternative pathway [47]. This was emphasized in a xenogeneic model involving the exposition of human blood to modified porcine endothelial cells [48]. In parallel, CR1 was here the only underrepresented protein in the complement regulators category during caABMR, and was strongly and negatively correlated with glomerulitis. In normal glomeruli, CR1 is known to be exclusively expressed in podocytes [49]. One hypothesis to explain this underrepresentation could be a loss of podocytes occurring in transplant glomerulopathy, further supported by the underrepresentation of the 2 podocyte proteins nephrin (NPHS1) and podocalyxin (PODXL) in caABMR. This podocyte loss would be in accordance with clinical proteinuria, classically associated with transplant glomerulopathy, and histological lesions of focal segmental glomerulosclerosis, frequently superimposed on advanced chronic glomerular lesions.

A recent transcriptomic and immunohistochemical study highlighted caveolin-1 as potential marker of chronic ABMR, found on the endothelium surface of both glomeruli and peritubular capillaries [50]. Herein we did not detect caveolin-1 by mass spectrometry, and so our findings could not support this result.

We described for the first time the ECM proteome alterations during transplant glomerulopathy. As expected, many overrepresented proteins were core matrisome components, but also many were ECM-regulator proteins, partly involved in the coagulation system, that give clues to potentially targetable proteins to prevent extracellular matrix expansion. We could notably notice 4 modulated collagens, with the overrepresentation of COL15A1 and COL12A1 and the underrepresentation of COL4A6 and COL1A1. Collagen XV, a basement membrane-associated collagen notably known to be expressed in microcirculation tissue such as placenta [51], was the second most correlated protein with the glomerular chronicity score cg + mm, and thus could be a relevant marker of glomerular matrix remodeling in kidney transplants.

We did not reproduce the results of Clotet-Freixas et al., considering the underrepresentation of ECM proteins during aABMR [12]. In addition to a lack of statistical power, two explanations could be hypothesized: differences considering time post-transplantation and the control groups. Clotet-Freixas et al., analyzed early aABMR cases compared to acute tubular necrosis and acute cellular rejection, all within the first 3 months post-transplantation, while we mainly analyzed late aABMR with one-year protocol biopsies as controls. We could hypothesize that biopsies at one year already display multifactorial modification of their ECM (healing of ischemia-reperfusion injuries, calcineurin inhibitor toxicity) that could mask the modification occurring during aABMR.

Our study has some limitations. The proteomic analysis concerned a small cohort of highly selected cases with no differential diagnosis other than stable graft controls for comparison. Still, our ABMR cohort is the largest one described to date through proteomic analysis and it was our goal for this first study to carefully select prototypic cases. The main limitation in our proteome characterization of transplant glomerulopathy is probably that all analyzed cases were chronic ABMR with activity. Together with the disparities in the C4d status of ABMR between caABMR (5 C4d positive) and aABMR (2 C4d positive), one could argue that it could at least partially explain the strong influence of the complement system in our protein profile of transplant glomerulopathy. Still, according to the Banff scheme, the C4d status of ABMR is assessed on peritubular capillaries, not in the glomeruli, that were exclusively analyzed here. Moreover, an enhanced C4d expression by immunohistochemistry in transplant glomerulopathy compared to glomeruli without chronicity, as seen in our cohort (Table 1), is classically seen and in accordance with the literature [52,53]. Finally, we are also aware that our study could not strictly distinguish immune-related glomerular proteome modifications from non-immune-related ones such as the influence of chronic exposure to immunosuppressive agents such as calcineurin inhibitors. However, all cases of transplant glomerulopathy were here at least attributable to an antibody-mediated process.

To conclude, by focusing on the glomerular proteome modifications of ABMR in human kidney transplantation, this study brings novel insights into glomerulitis and transplant glomerulopathy. We highlighted several cytokine-mediated targetable pathways as well as immunomarkers of potential diagnostic interest. Moreover, this study stresses the involvement of the complement system and of coagulation in transplant glomerulopathy and brings a thorough description of the resultant remodeling of the extracellular matrix. We hope that this study will pave the way for the discovery of innovative diagnostic biomarkers and new therapeutic strategies in ABMR.

## Figures and Tables

**Table 1:** Demographical characteristics of the cohort.

	aABMR Group (*n* = 11)		caABMR Group (*n* = 10)		Stable Graft Group (*n* = 8)	
	*n*		*n*		*n*	
Recipients						
Age at the time of biopsy, year, median (IQR)	11	61 (53–68)	10	59 (50–71)	8	50 (36–67)
Male, *n* (%)	11	8 (72.7)	10	7 (70)	8	5 (63)
ESRD causes, *n* (%)	11		10		8	
Glomerulonephritis		4 (36.4)		2 (20)		1 (13)
Diabetes		0 (0)		0 (0)		0 (0)
Polycystic kidney disease		2 (18.2)		4 (40)		3 (38)
Tubulo-interstitial disease		2 (18.2)		2 (20)		2 (25)
Vascular nephropathy		1 (9.1)		1 (10)		0 (0)
Unknown		2 (18.2)		1 (10)		1 (13)
Others		0 (0)		0 (0)		1 (13)
Prior transplantation, *n* (%)	11	6 (54.5)	10	1 (10)	8	2 (25)
Donors						
Age, years, median (IQR)	10	54 (49–62)	6	63 (57–64)	6	44 (34–60)
Men, *n* (%)	9	2 (22.2)	6	5 (83.3)	7	3 (43)
Living donor, *n* (%)	11	3 (27.3)	8	0 (0)	7	3 (43)
Anti-HLA antibody at the time of transplantation	9		4		8	
No evidence of anti-HLA antibodies, *n* (%)		1 (11.1)		3 (75)		6 (75)
Evidence of anti-HLA antibodies but not DSA, *n* (%)		1 (11.1)		1 (25)		2 (25)
DSA, *n* (%)		7 (77.8)		0 (0)		0 (0)
DSA at the time of ABMR diagnosis	11		10		8	
DSA, *n* (%)		11 (100)		10 (100)		0 (0)
Class I DSA, *n* (%)		3 (27)		2 (20)		0 (0)
Class II DSA, *n* (%)		10 (91)		8 (80)		0 (0)
*De novo* DSA, *n* (%)		4 (36)		10 (100)		0 (0)
Clinical and biological parameters at the time of biopsy						
Months post-transplantation of biopsy, median (IQR)	11	9 (3–28)	10	77 (45–126)	8	12 (12–13)
Biopsy indications	11		10		8	
For cause, *n* (%)		6 (55)		8 (80)		0 (0)
Protocol, *n* (%)		5 (45)		2 (20)		8 (100)
eGFR at diagnosis, mL/min/1.73 m^2^, median (IQR)	11	42 (35–53)	10	22 (16–35)	8	76.5 (52–82)
Proteinuria at diagnosis, mg/mmol, median (IQR)	10	48 (24–110)	9	94 (88–241)	8	10 (8–14)
Immunosuppression at the time of biopsy, *n* (%)	11		10		8	
Tacrolimus		8 (73)		6 (60)		6 (75)
Cyclosporin A		2 (18)		4 (40)		2 (25)
Everolimus		3 (27)		1 (10)		1 (13)
Azathioprine		0 (0)		1 (10)		1 (13)
Mycophenolic acid		9 (82)		7 (70)		6 (75)
Corticosteroids		10 (91)		6 (60)		5 (63)
Tacrolimus and mycophenolic acid		8 (73)		5 (50)		4 (50)
Histological parameters						
Total number of glomeruli, median (IQR)	11	15 (11–18)	10	20.5 (17–22.75)	8	16 (15–18)
Number of globally sclerotic glomeruli, median (IQR)	11	1 (0–1)	10	1 (0.25–7)	8	0 (0–0.3)
Banff scoring			10			
g, mean (SD)	11	1.6 (0.7)		1.8 (0.4)	8	0 (0)
ptc, mean (SD)	11	1.5 (0.8)		2.1 (0.7)	8	0 (0)
cg, mean (SD)	11	0 (0)		2.3 (0.8)	8	0 (0)
mm, mean (SD)	11	0.1 (0.3)		1.6 (0.7)	8	0 (0)
i, mean (SD)	11	0.6 (0.8)		0.1 (0.3)	8	0 (0)
t, mean (SD)	11	0.5 (1)		0.1 (0.3)	8	0 (0)
v, mean (SD)	10	0.2 (0.6)		0 (0)	6	0 (0)
ci, mean (SD)	11	0.9 (0.5)		1.7 (0.7)	8	0.6 (0.7)
ct, mean (SD)	11	0.9 (0.5)		1.7 (0.7)	8	0.6 (0.7)
ah, mean (SD)	11	0.1 (0.3)		2 (1.3)	8	0.8 (0.9)
cv, mean (SD)	10	0.7 (0.8)		1.9 (1.1)	6	1.2 (1)
C4d positivity on peritubular capillaries, *n* (%)	11	2 (18)		5 (50)	7	0 (0)
C4d positivity in the glomeruli, mean (SD)	11	0.8 (1.0)	10	1.9 (1.2)	7	0.3 (0.5)

Abbreviations: aABMR, active antibody-mediated rejection; caABMR, chronic active antibody-mediated rejection; IQR, interquartile range; ESRD, end-stage renal disease; HLA, human leukocyte antigens; DSA, donor-specific antibodies; eGFR, estimated glomerular filtration rate; MFI, mean fluorescence intensity.

**Table 2:** Top-25 proteins differentially represented between active ABMR, chronic active ABMR and stable graft.

aABMR vs. SG				caABMR vs. SG			
UniProt Entry	Protein Name	Fold-Change	Adjusted *p*-Value	UniProt Entry	Protein Name	Fold-Change	Adjusted *p*-Value
P19971	TYMP	3.51	1.10 × 10^−3^	E9PF17	VCAN	14.62	7.07 × 10^−4^
P32455	GBP1	3.48	1.10 × 10^−3^	A0A0A0MS41	SFXN3	9.90	7.07 × 10^−4^
P31146	CORO1A	3.73	1.10 × 10^−3^	Q6PCB0	VWA1	5.39	7.07 × 10^−4^
P23381	WARS	2.33	1.10 × 10^−3^	A0A087X0K0	COL15A1	5.09	7.07 × 10^−4^
A0A087X1Z3	PSME2	2.00	1.10 × 10^−3^	Q96C19	EFHD2	4.88	7.07 × 10^−4^
P28062	PSMB8	1.67	1.10 × 10^−3^	P21589	NT5E	4.68	7.07 × 10^−4^
Q9UJW2	TINAG	0.45	1.10 × 10^−3^	P19971	TYMP	4.17	7.07 × 10^−4^
Q96C19	EFHD2	5.20	1.93 × 10^−3^	P55884	EIF3B	3.07	7.07 × 10^−4^
Q9ULZ3	PYCARD	14.09	2.81 × 10^−3^	P43121	MCAM	2.96	7.07 × 10^−4^
G5E9W9	GIMAP4	2.28	2.81 × 10^−3^	Q96CX2	KCTD12	2.74	7.07 × 10^−4^
Q16401	PSMD5	2.00	2.81 × 10^−3^	O60506	SYNCRIP	2.72	7.07 × 10^−4^
P13796	LCP1	2.61	4.16 × 10^−3^	P23381	WARS	2.57	7.07 × 10^−4^
P42224	STAT1	3.31	4.16 × 10^−3^	Q13596	SNX1	2.26	7.07 × 10^−4^
O14745	SLC9A3R1	0.59	5.15 × 10^−3^	A0A1B0GVU9	QARS	2.02	7.07 × 10^−4^
P28838	LAP3	2.08	5.15 × 10^−3^	P41218	MNDA	1.74	7.07 × 10^−4^
H7C0J5	CEP104	0.21	5.15 × 10^−3^	P04083	ANXA1	1.56	7.07 × 10^−4^
Q9UJ70	NAGK	1.84	5.15 × 10^−3^	P49411	TUFM	0.65	7.07 × 10^−4^
A2ACR1	PSMB9	1.92	5.15 × 10^−3^	A2A274	ACO2	0.51	7.07 × 10^−4^
P29508	SERPINB3	0.53	7.34 × 10^−3^	Q9BQI0	AIF1L	0.48	7.07 × 10^−4^
A0A0G2JMH6	HLA-DRA	1.61	7.34 × 10^−3^	Q9Y2S2	CRYL1	0.44	7.07 × 10^−4^
A0A087X1J7	GPX3	0.53	1.10 × 10^−2^	P00918	CA2	0.41	7.07 × 10^−4^
P04040	CAT	0.50	1.34 × 10^−2^	Q93088	BHMT	0.41	7.07 × 10^−4^
H3BM42	GLG1	7.13	1.34 × 10^−2^	A0A1B0GU86	ACY1	0.37	7.07 × 10^−4^
A0A0A0MSV9	TAPBP	3.41	1.34 × 10^−2^	Q96DG6	CMBL	0.35	7.07 × 10^−4^
Q9Y3Z3	SAMHD1	2.26	1.34 × 10^−2^	A0A087X1J7	GPX3	0.32	7.07 × 10^−4^

Non-parametric Mann-Whitney U tests were performed to compare the protein expressions between groups. *p*-values were secondarily adjusted according to the Benjamini-Hochberg correction. A fold change above 1.5 implies a significant overrepresentation of the protein, whereas a ratio below 0.66 an underrepresentation. Abbreviations: aABMR, active antibody-mediated rejection; caABMR, chronic active antibody-mediated rejection; SG, stable graft control.

**Table 3:** Top-10 proteins positively correlated between their abundances by mass spectrometry and the Banff morphological glomerulitis score.

Correlation with Glomerulitis Score			
UniProt Access	Protein	Spearman’s Coefficient	Adjusted *p*-Value
P31146	CORO1A	0.92	1.16 × 10^−9^
P13796	LCP1	0.91	5.60 × 10^−9^
Q96C19	EFHD2	0.86	7.30 × 10^−7^
Q9Y3Z3	SAMHD1	0.86	1.11 × 10^−6^
Q9ULZ3	PYCARD	0.85	1.94 × 10^−6^
P32455	GBP1	0.84	2.00 × 10^−6^
P23381	WARS	0.84	2.11 × 10^−6^
P19971	TYMP	0.83	3.62 × 10^−6^
P43121	MCAM	0.76	1.09 × 10^−4^
A0A0A0MSV9	TAPBP	0.75	1.45 × 10^−4^

From the 77 proteins deregulated in glomerulitis, Spearman’s correlation analyzes were performed between proteins abundance by mass spectrometry and the glomerulitis semi-quantitative g score. According to the Banff rules, the g score is graded as such: 0 (no glomerulitis), 1 (glomerulitis in <25% of the glomeruli), 2 (25–75%), 3 (glomerulitis in >75% of the glomeruli).

**Table 4:** Top-25 proteins differentially represented in chronic active ABMR compared to active ABMR.

caABMR vs. aABMR			
UniProt Entry	Protein Name	Fold-Change	Adjusted *p*-Value
P08603	CFH	2.83	3.80 × 10^−3^
A0A3B3IU24	HTRA1	7.56	6.36 × 10^−3^
A0A286YEY1	IGHA1	3.29	6.36 × 10^−3^
A0A0S2Z4L3	PROS1	13.80	6.36 × 10^−3^
A0A087X0K0	COL15A1	3.89	6.36 × 10^−3^
P55884	EIF3B	2.61	6.36 × 10^−3^
Q08431	MFGE8	2.00	6.36 × 10^−3^
O60506	SYNCRIP	1.77	6.36 × 10^−3^
B1ALD9	POSTN	3.84	6.36 × 10^−3^
Q6PCB0	VWA1	2.55	6.36 × 10^−3^
P05141	SLC25A5	0.60	6.36 × 10^−3^
P02652	APOA2	2.23	6.36 × 10^−3^
B7ZKJ8	ITIH4	2.60	6.36 × 10^−3^
P01008	SERPINC1	2.12	6.36 × 10^−3^
Q9BXR6	CFHR5	2.70	6.36 × 10^−3^
P21589	NT5E	2.84	6.36 × 10^−3^
P00747	PLG	1.84	6.36 × 10^−3^
P14550	AKR1A1	0.65	6.36 × 10^−3^
Q9UH99	SUN2	7.34	6.36 × 10^−3^
A0A0J9YY99	Uncharacterized	9.16	8.38 × 10^−3^
A0A3B3ISR2	C1R	2.67	8.38 × 10^−3^
O75368	SH3BGRL	6.17	9.78 × 10^−3^
P01624	IGKV3-15	4.29	9.78 × 10^−3^
P0DOY2	IGLC2	2.46	9.78 × 10^−3^
P05155	SERPING1	1.75	9.78 × 10^−3^

Non-parametric Mann-Whitney U tests were performed to compare the protein expressions between groups. *p*-values were secondarily adjusted according to the Benjamini-Hochberg correction. A fold change above 1.5 implies a significant overrepresentation of the protein, whereas a ratio below 0.66 an underrepresentation. Abbreviations: aABMR, active antibody-mediated rejection; caABMR, chronic active antibody-mediated rejection; SG, stable graft control.

**Table 5:** Top-10 proteins positively correlated between their abundances by mass spectrometry and the Banff morphological scores cg + mm of chronic glomerular injuries.

Correlation with Transplant Glomerulopathy (cg + mm Scores)			
UniProt Access	Protein	Spearman’s Coefficient	Adjusted *p*-Value
P08603	CFH	0.90	4.47 × 10^−5^
A0A087X0K0	COL15A1	0.88	7.86 × 10^−5^
B1ALD9	POSTN	0.82	2.64 × 10^−3^
Q6PCB0	VWA1	0.81	2.73 × 10^−3^
P02652	APOA2	0.79	4.82 × 10^−3^
P04003	C4BPA	0.78	4.82 × 10^−3^
A0A0S2Z4L3	PROS1	0.78	5.24 × 10^−3^
A0A3B3IU24	HTRA1	0.77	5.59 × 10^−3^
A0A286YEY1	IGHA1	0.77	5.63 × 10^−3^
P01008	SERPINC1	0.76	5.85 × 10^−3^

From the 137 protein dataset defining chronic antibody-mediated injuries, Spearman’s correlation analyzes were performed with the sum of the Banff chronic glomerular injury scores, i.e., double contours (cg) and mesangial expansion (mm), both graded from 0 to 3.

## Data Availability

The proteomic data that support the findings of this study are openly available in the ProteomeXchange Consortium via the PRIDE [31] partner repository with the dataset identifier PXD021852.

## Supplementary Materials

The following supporting information can be downloaded at: https://www.mdpi.com/article/10.3390/biomedicines10030569/s1, Supplemental Methods (laser microdissection, sample preparation for mass spectrometry, proteomic analysis, immunohistochemical analysis); Table S1: List of the 77 proteins differentiating active antibody-mediated glomerular injuries from stable grafts, in ascending order of adjusted *p*-values; Table S2: List of the 335 proteins differentiating chronic active antibody-mediated glomerular injuries from stable grafts, in ascending order of adjusted *p*-values; Table S3: Detailed histological scores and relative protein abundances of the five antibodies tested by immunohistochemistry for each analyzed case; Table S4: List of the 137 proteins differentiating chronic active from active antibody-mediated glomerular injuries, in ascending order of adjusted *p*-values; Table S5: List of the 135 extracellular matrix proteins of this study according to the Matrisome Project database; Table S6: Abundance modifications of selected extracellular matrix and podocyte-specific proteins in transplant glomerulopathy; Figure S1: Flow-chart of the study; Figure S2: Fold changes of the 59 proteins differentially represented in both antibody-mediated rejection groups compared to the stable grafts; Figure S3: Dot plots showing protein abundances by mass spectrometry depending on the glomerulitis score; Figure S4: Box plots showing protein abundances by mass spectrometry depending on the C4d status for the 5 proteins tested by immunohistochemistry; Figure S5: Dot plots showing protein abundances by mass spectrometry depending on the cg+mm score, reflecting changes seen in transplant glomerulopathy.
