# Supplementary material for: The Proteome of Antibody-Mediated Rejection: From Glomerulitis to Transplant Glomerulopathy
Source: Biomedicines. 2022 Feb 28;10(3):569. doi: 10.3390/biomedicines10030569 (PMC8945687; doi:10.3390/biomedicines10030569)
Supplement: Supplementary file 1 [file biomedicines-10-00569-s001.zip › biomedicines-1607401-supplementary.pdf]

Supplemental information

TABLE OF CONTENTS

1. Supplemental Methods

1.1 Laser microdissection
1.2 Protein extraction and sample preparation for mass spectrometry
1.3 Proteomic analysis
1.4 Immunohistochemical analysis
1.5 References

2. Supplemental Tables and Figures

Supplemental Table S1. List of the 77 proteins differentiating active antibody-mediated glomerular injuries from stable grafts, in ascending order of adjusted p-values

Supplemental Table S2. List of the 335 proteins differentiating chronic active antibody-mediated glomerular injuries from stable grafts, in ascending order of adjusted p-values

Supplemental Table S3. Detailed histological scores and relative protein abundances of the five antibodies tested by immunohistochemistry for each analyzed case

Supplemental Table S4. List of the 137 proteins differentiating chronic active from active antibody-mediated glomerular injuries, in ascending order of adjusted p-values

Supplemental Table S5. List of the 135 extracellular matrix proteins of this study according to the Matrisome Project database

Supplemental Table S6. Abundance modifications of selected extracellular matrix and podocyte-specific proteins in transplant glomerulopathy

Supplemental Figure S1. Flow-chart of the study

Supplemental Figure S2. Fold changes of the 59 proteins differentially represented in both antibody-mediated rejection groups compared to the stable grafts.

Supplemental Figure S3. Dot plots showing protein abundances by mass spectrometry depending on the glomerulitis score.

Supplemental Figure S4. Box plots showing protein abundances by mass spectrometry depending on the C4d status for the 5 proteins tested by immunohistochemistry.

Supplemental Figure S5. Dot plots showing protein abundances by mass spectrometry depending on the cg+mm score, reflecting changes seen in transplant glomerulopathy.

1. Supplemental Methods

1.1 Laser microdissection

Biopsy samples were already fixed in 4% acetic formalin and paraffin-embedded, as tissue sections were routinely processed for diagnosis purposes. For each sample, 5 µm thick sections were performed and deposited on PEN Membrane Glass Slides (Thermo Fisher Scientific, California, USA). The sections were then dewaxed, rehydrated and stained with hematoxylin. For each replicate, 50 non globally sclerotic glomerular sections were isolated using the PALM MicroBeam (Zeiss) laser microdissector. Two replicates were performed for each biopsy, and a total of nine replicates were performed for a normal kidney (surgical specimen), used as calibrator.

1.2 Protein extraction and sample preparation for mass spectrometry

The steps of sample preparation and protein digestion were performed as previously described [1]. Microdissected tissues were incubated in a Tris-HCl pH 6.8 solution for 2 hours at 95°C, including a sonication stage. Samples were loaded on a 10% acrylamide SDS-PAGE gel. Migration was stopped when the samples entered the resolving gel and the proteins were visualized by colloidal blue staining. Each band of gel was then digitized, and the optical density was measured to allow a standardization of the protein amount before mass spectrometry. Each SDS-PAGE band was cut into 1 mm x 1 mm gel pieces. Gel pieces were destained in 25 mM ammonium bicarbonate (NH_4HCO_3), 50% acetonitrile (ACN) and shrunk in ACN for 10 minutes. After ACN removal, the gel pieces were dried at room temperature. The proteins were first reduced in 10 mM dithiothreitol, 100 mM NH_4HCO_3 for 60 minutes at 56°C then alkylated in 100 mM iodoacetamide, 100 mM NH_4HCO_3 for 60 minutes at room temperature and shrunk in ACN for 10 minutes. After ACN removal, the gel pieces were rehydrated with 100 mM NH_4HCO_3 for 10 minutes at room temperature. Before protein digestion, the gel pieces were shrunk in ACN for 10 minutes and dried at room temperature. The proteins were digested by incubating each gel slice with 10 ng/µL of trypsin (T6567, Sigma-Aldrich) in 40 mM NH_4HCO_3, 10% ACN, rehydrated at 4°C for 10 minutes, and were finally incubated overnight at 37°C. The resulting peptides were extracted from the gel in three steps: the first incubation was in 40 mM NH_4HCO_3, 10% ACN for 15 minutes at room temperature and two subsequent incubations were in 47.5 % ACN, 5% formic acid for 15 minutes at room temperature. The three collected extractions were pooled with the initial digestion supernatant, dried in a SpeedVac, and re-suspended in 0.1% formic acid before nanoLC-MS/MS analysis.

1.3 Proteomic analysis

Mass Spectrometry-based proteomic analysis of the samples was performed as described previously [1]. NanoLC-MS/MS analysis was performed using an Ultimate 3000 RSLC Nano-UPHLC system (Thermo Scientific, USA) coupled to a nanospray Orbitrap Fusion™ Lumos™ Tribrid™ Mass Spectrometer (Thermo Fisher Scientific, California, USA). Each peptide extracts were loaded on a 300 µm ID x 5 mm PepMap C18 precolumn (Thermo Scientific, USA) at a flow rate of 10 µL/min. After a 3 min desalting step, peptides were separated on a 50 cm EasySpray column (75 µm ID, 2 µm C18 beads, 100 Å pore size, ES803, Thermo Fisher Scientific) with a 4-40% linear gradient of solvent B (0.1% formic acid in 80% ACN) in 55 min. The separation flow rate was set at 300 nL/min. The mass spectrometer operated in positive ion mode at a 2.0 kV needle voltage. Data was acquired using Xcalibur 4.1 software in a data-dependent mode. MS scans (m/z 375-1500) were recorded at a resolution of R=120000 (@ m/z 200) and an AGC target of $4x10^5$ ions collected within 50 ms, followed by a top speed duty cycle of up to 3 seconds for MS/MS acquisition. Precursor ions (2 to 7 charge states) were isolated in the quadrupole with a mass window of 1.6 Th and fragmented with HCD@30% normalized collision energy. MS/MS data was acquired in the Orbitrap cell with a resolution of R=30000 (@m/z 200), AGC target of $5x10^4$ ions and a maximum injection time of 100 ms. Selected precursors were excluded for 60 seconds. For protein identification, Mascot 2.5 algorithm through Proteome Discoverer 1.4 Software (Thermo Fisher Scientific Inc.) was used in batch mode by searching against the UniProt Homo sapiens database (73 658 entries, Reference Proteome Set, release date: December 13, 2018) from http://www.uniprot.org/ website. Two missed enzyme cleavages were allowed. Mass tolerances in MS and MS/MS were set to 10 ppm and 0.02 Da. Oxidation of methionine, acetylation of lysine and deamidation of asparagine and glutamine were searched as dynamic modifications. Carbamidomethylation on cysteine was searched as static modification. Raw LC-MS/MS data were imported in Proline Studio [2] for feature detection, alignment, and quantification. Proteins identification was accepted only with at least 2 specific peptides with a pretty rank=1 and with a protein FDR value less than 1.0% calculated using the “decoy” option in Mascot. Label-free quantification of MS1 level by extracted ion chromatograms (XIC) was carried out with parameters indicated previously [1]. The normalization was carried out on median of ratios. The inference of missing values was applied with 5% of the background noise. The mass spectrometry proteomics data have been deposited to the ProteomeXchange Consortium via the PRIDE [3] partner repository with the dataset identifier PXD021852.

1.4 Immunohistochemical analysis

For immunohistochemistry, 2.5µm thick sections were performed, dewaxed and rehydrated. Antigen retrieval was performed in a 1mM Tris-EDTA pH=9 solution. All staining procedures were performed in an automated autostainer (Dako-Agilent, Santa Clara, United States) using standard reagents provided by the manufacturer. Five commercial primary antibodies were used, targeting thymidine phosphorylase (TYMP), tryptophan--tRNA ligase, cytoplasmic (WARS1), coronin-1A (CORO1A), guanylate-binding protein 1 (GBP1), EF-hand domain-containing protein D2 (EFHD2). The sections were incubated with the corresponding antibody for 45 min at room temperature. EnVision Flex/horseradish peroxidase (Dako-Agilent) was used for signal amplification, revealed by 3,3'-diamino-benzidine (Dako-Agilent). The slides were counterstained with hematoxylin, dehydrated and mounted. Each immunohistochemical run contained a negative (buffer, no primary antibody) and positive control (graft nephrectomy with chronic active ABMR lesions).

Target	Manufacturer	Host species	Clonality	Dilution
Tryptophanyl tRNA synthetase/WARS1	Abcam	Rabbit	Monoclonal EPR3423	1:3000
Coronin1a/TACO	Abcam	Rabbit	Monoclonal EPR19467-36	1:3000
Thymidine Phosphorylase/TYMP	Abcam	Mouse	Monoclonal P-GF.44C	1:200
EF-hand domain family member 2/EFHD2	Sigma-Aldrich	Rabbit	Polyclonal	1:200
Guanylate-binding protein 1/GBP1	Abcam	Mouse	Monoclonal OTI1B2	1:50

1.5 References

1. Henriet, E.; Hammoud, A.A.; Dupuy, J.-W.; Dartigues, B.; Ezzoukry, Z.; Dugot-Senant, N.; Leste-Lasserre, T.; Pallares-Lupon, N.; Nikolski, M.; Le Bail, B.; et al. Argininosuccinate Synthase 1 (ASS1): A Marker of Unclassified Hepatocellular Adenoma and High Bleeding Risk. *Hepatology* 2017, doi:10.1002/hep.29336.
2. Bouyssié, D.; Hesse, A.-M.; Mouton-Barbosa, E.; Rompais, M.; Macron, C.; Carapito, C.; Gonzalez de Peredo, A.; Couté, Y.; Dupierris, V.; Burel, A.; et al. Proline: An Efficient and User-Friendly Software Suite for Large-Scale Proteomics. *Bioinformatics* 2020, *36*, 3148–3155, doi:10.1093/bioinformatics/btaa118.
3. Perez-Riverol, Y.; Csordas, A.; Bai, J.; Bernal-Llinares, M.; Hewapathirana, S.; Kundu, D.J.; Inuganti, A.; Griss, J.; Mayer, G.; Eisenacher, M.; et al. The PRIDE Database and Related Tools and Resources in 2019: Improving Support for Quantification Data. *Nucleic Acids Res.* 2019, *47*, D442–D450, doi:10.1093/nar/gky1106.
4. Clotet-Freixas, S.; McEvoy, C.M.; Batruch, I.; Pastrello, C.; Kotlyar, M.; Van, J.A.D.; Arambewela, M.; Boshart, A.; Farkona, S.; Niu, Y.; et al. Extracellular Matrix Injury of Kidney Allografts in Antibody-Mediated Rejection: A Proteomics Study. *J. Am. Soc. Nephrol.* 2020, doi:10.1681/ASN.2020030286.

2. Supplemental Tables and Figures

Supplemental Table S1. List of the 77 proteins differentiating active antibody-mediated glomerular injuries from stable grafts, in ascending order of adjusted p-values

UniProt access	Protein name	Corresponding gene name	Fold-change aABMR/SG	Adjusted p-value
P19971	Thymidine phosphorylase	TYMP	3.51	1.10E-03
P32455	Guanylate-binding protein 1	GBP1	3.48	1.10E-03
P31146	Coronin-1A	CORO1A	3.73	1.10E-03
P23381	Tryptophan--tRNA ligase, cytoplasmic	WARS1	2.33	1.10E-03
A0A087X1Z3	Proteasome activator complex subunit 2	PSME2	2.00	1.10E-03
P28062	Proteasome subunit beta type-8	PSMB8	1.67	1.10E-03
Q9UJW2	Tubulointerstitial nephritis antigen	TINAG	0.45	1.10E-03
Q96C19	EF-hand domain-containing protein D2	EFHD2	5.20	1.93E-03
Q9ULZ3	Apoptosis-associated speck-like protein containing a CARD	PYCARD	14.09	2.81E-03
G5E9W9	GTPase IMAP family member 4	GIMAP4	2.28	2.81E-03
Q16401	26S proteasome non-ATPase regulatory subunit 5	PSMD5	2.00	2.81E-03
P13796	Plastin-2	LCP1	2.61	4.16E-03
P42224	Signal transducer and activator of transcription 1-alpha/beta	STAT1	3.31	4.16E-03
O14745	Na(+)/H(+) exchange regulatory cofactor NHE-RF1	SLC9A3R1	0.59	5.15E-03
P28838	Cytosol aminopeptidase	LAP3	2.08	5.15E-03
H7C0J5	Centrosomal protein of 104 kDa	CEP104	0.21	5.15E-03
Q9UJ70	N-acetyl-D-glucosamine kinase	NAGK	1.84	5.15E-03
A2ACR1	Proteasome subunit beta	PSMB9	1.92	5.15E-03
P29508	Serpin B3	SERPINB3	0.53	7.34E-03
A0A0G2JMH6	HLA class II histocompatibility antigen, DR alpha chain	HLA-DRA	1.61	7.34E-03
A0A087X1J7	Glutathione peroxidase	GPX3	0.53	1.10E-02
P04040	Catalase	CAT	0.50	1.34E-02
H3BM42	Golgi apparatus protein 1, isoform CRA_c	GLG1	7.13	1.34E-02
A0A0A0MSV9	Tapasin	TAPBP	3.41	1.34E-02
Q9Y3Z3	Deoxynucleoside triphosphate triphosphohydrolase SAMHD1	SAMHD1	2.26	1.34E-02
P52907	F-actin-capping protein subunit alpha-1	CAPZA1	1.91	1.34E-02
J3QLE5	Small nuclear ribonucleoprotein-associated protein N (Fragment)	SNRPN	1.84	1.67E-02
P29590	Protein PML	PML	1.57	1.67E-02
P50440	Glycine amidinotransferase, mitochondrial	GATM	0.47	1.67E-02
Q86YZ3	Hornerin	HRNR	0.46	1.67E-02
F8VW96	Cysteine and glycine-rich protein 2	CSRP2	0.58	1.67E-02
Q10589	Bone marrow stromal antigen 2	BST2	3.25	2.25E-02
Q9Y6W5	Wiskott-Aldrich syndrome protein family member 2	WASF2	4.12	2.25E-02
P62136	Serine/threonine-protein phosphatase PP1-alpha catalytic subunit	PPP1CA	1.71	2.69E-02
Q13596	Sorting nexin-1	SNX1	1.69	2.69E-02
P09210	Glutathione S-transferase A2	GSTA2	0.46	2.69E-02
P28906	Hematopoietic progenitor cell antigen CD34	CD34	0.64	2.69E-02
P14902	Indoleamine 2,3-dioxygenase 1	IDO1	2.85	2.69E-02
I3L0K7	Heat shock protein 75 kDa, mitochondrial	TRAP1	1.94	2.69E-02
O76041	Nebulette	NEBL	0.31	2.79E-02

F8VY04	Adenylate kinase 2, mitochondrial	AK2	0.54	2.79E-02
E9PF17	Versican core protein	VCAN	9.34	2.79E-02
P05164	Myeloperoxidase	MPO	1.67	2.79E-02
P05198	Eukaryotic translation initiation factor 2 subunit 1	EIF2S1	3.51	2.79E-02
P13716	Delta-aminolevulinic acid dehydratase	ALAD	0.57	2.79E-02
E9PHS0	Glutathione S-transferase LANCL1 (Fragment)	LANCL1	3.04	2.79E-02
J3KQL8	Apolipoprotein L2	APOL2	2.13	2.79E-02
Q14651	Plastin-1	PLS1	1.92	2.79E-02
P52943	Cysteine-rich protein 2	CRIP2	0.57	2.79E-02
P61626	Lysozyme C	LYZ	0.53	2.79E-02
H0YJW3	Alpha-actinin-1 (Fragment)	ACTN1	0.54	2.79E-02
P12004	Proliferating cell nuclear antigen	PCNA	2.94	2.79E-02
J3KT73	60S ribosomal protein L38	RPL38	1.60	3.06E-02
P26447	Protein S100-A4	S100A4	2.54	3.06E-02
A0A096LNZ9	Ubiquitin-like protein ISG15 (Fragment)	ISG15	3.75	3.06E-02
P43121	Cell surface glycoprotein MUC18	MCAM	1.84	3.06E-02
Q06323	Proteasome activator complex subunit 1	PSME1	1.62	3.06E-02
A0A2R8Y7G9	Uncharacterized protein	H3Y1	2.93	3.06E-02
Q13185	Chromobox protein homolog 3	CBX3	6.38	3.06E-02
A0A0G2JMX7	Microtubule-associated protein	MAPT	0.47	3.06E-02
Q08554	Desmocollin-1	DSC1	0.51	3.06E-02
Q96JY6	PDZ and LIM domain protein 2	PDLIM2	0.65	3.06E-02
J3QT28	Mitotic checkpoint protein BUB3 (Fragment)	BUB3	5.25	3.06E-02
E9PBF6	Lamin-B1	LMNB1	1.73	3.06E-02
A8MW49	Fatty acid-binding protein, liver	FABP1	0.54	3.69E-02
Q9NZN3	EH domain-containing protein 3	EHD3	0.62	3.69E-02
A0A087WUV8	Basigin	BSG	1.64	3.69E-02
A0A0A0MS41	Sideroflexin	SFXN3	5.74	3.69E-02
P05362	Intercellular adhesion molecule 1	ICAM1	1.59	3.69E-02
P12273	Prolactin-inducible protein	PIP	0.55	3.69E-02
Q8N3V7	Synaptopodin	SYNPO	0.60	3.69E-02
P69905	Hemoglobin subunit alpha	HBA1	0.51	4.46E-02
P17900	Ganglioside GM2 activator	GM2A	0.47	4.46E-02
Q9Y6K5	2'-5'-oligoadenylate synthase 3	OAS3	4.81	4.46E-02
Q9UJY1	Heat shock protein beta-8	HSPB8	2.46	4.46E-02
O43301	Heat shock 70 kDa protein 12A	HSPA12A	0.64	4.46E-02
Q01469	Fatty acid-binding protein 5	FABP5	0.49	4.46E-02

Non-parametric Mann-Whitney tests were performed to compare the protein expressions between the active antibody-mediated rejection (aABMR) and stable graft (SG) group. P-values were secondarily adjusted according to the Benjamini-Hochberg correction. Of note, two proteins were removed from this list (KRT9 and KRT2), being a contaminant from the epidermis. Abbreviations: aABMR, active antibody-mediated rejection; SG, stable graft control.

Supplemental Table S2. List of the 335 proteins differentiating chronic active antibody-mediated glomerular injuries from stable grafts, in ascending order of adjusted p-values

UniProt access	Protein name	Corresponding gene name	Fold-change caABMR/SG	Adjusted p-value
E9PF17	Versican core protein	VCAN	14.62	7.07E-04
A0A0A0MS41	Sideroflexin	SFXN3	9.90	7.07E-04
Q6PCB0	von Willebrand factor A domain-containing protein 1	VWA1	5.39	7.07E-04
A0A087X0K0	Collagen alpha-1(XV) chain	COL15A1	5.09	7.07E-04
Q96C19	EF-hand domain-containing protein D2	EFHD2	4.88	7.07E-04
P21589	5'-nucleotidase	NT5E	4.68	7.07E-04
P19971	Thymidine phosphorylase	TYMP	4.17	7.07E-04
P55884	Eukaryotic translation initiation factor 3 subunit B	EIF3B	3.07	7.07E-04
P43121	Cell surface glycoprotein MUC18	MCAM	2.96	7.07E-04
Q96CX2	BTB/POZ domain-containing protein KCTD12	KCTD12	2.74	7.07E-04
O60506	Heterogeneous nuclear ribonucleoprotein Q	SYNCRIP	2.72	7.07E-04
P23381	Tryptophan--tRNA ligase, cytoplasmic	WARS1	2.57	7.07E-04
Q13596	Sorting nexin-1	SNX1	2.26	7.07E-04
A0A1B0GVU9	Glutamine--tRNA ligase (Fragment)	QARS	2.02	7.07E-04
P41218	Myeloid cell nuclear differentiation antigen	MNDA	1.74	7.07E-04
P04083	Annexin A1	ANXA1	1.56	7.07E-04
P49411	Elongation factor Tu, mitochondrial	TUFM	0.65	7.07E-04
A2A274	Aconitate hydratase, mitochondrial	ACO2	0.51	7.07E-04
Q9BQI0	Allograft inflammatory factor 1-like	AIF1L	0.48	7.07E-04
Q9Y2S2	Lambda-crystallin homolog	CRYL1	0.44	7.07E-04
P00918	Carbonic anhydrase 2	CA2	0.41	7.07E-04
Q93088	Betaine--homocysteine S-methyltransferase 1	BHMT	0.41	7.07E-04
A0A1B0GU86	Aminoacylase-1	ACY1	0.37	7.07E-04
Q96DG6	Carboxymethylenebutenolidase homolog	CMBL	0.35	7.07E-04
A0A087X1J7	Glutathione peroxidase	GPX3	0.32	7.07E-04
P30038	Delta-1-pyrroline-5-carboxylate dehydrogenase, mitochondrial	ALDH4A1	0.31	7.07E-04
P17927	Complement receptor type 1	CR1	0.28	7.07E-04
Q6IB77	Glycine N-acyltransferase	GLYAT	0.25	7.07E-04
Q02252	Methylmalonate-semialdehyde dehydrogenase [acylating], mitochondrial	ALDH6A1	0.25	7.07E-04
Q9UJW2	Tubulointerstitial nephritis antigen	TINAG	0.24	7.07E-04
Q16822	Phosphoenolpyruvate carboxykinase [GTP], mitochondrial	PCK2	0.18	7.07E-04
P09210	Glutathione S-transferase A2	GSTA2	0.17	7.07E-04
P62857	40S ribosomal protein S28	RPS28	17.97	9.05E-04
P13796	Plastin-2	LCP1	4.61	9.05E-04
P41091	Eukaryotic translation initiation factor 2 subunit 3	EIF2S3	2.52	9.05E-04
Q14112	Nidogen-2	NID2	2.40	9.05E-04
P36542	ATP synthase subunit gamma, mitochondrial	ATP5F1C	0.63	9.05E-04
P13804	Electron transfer flavoprotein subunit alpha, mitochondrial	ETFA	0.59	9.05E-04
P07195	L-lactate dehydrogenase B chain	LDHB	0.58	9.05E-04
P14550	Aldo-keto reductase family 1 member A1	AKR1A1	0.56	9.05E-04
P00367	Glutamate dehydrogenase 1, mitochondrial	GLUD1	0.54	9.05E-04
Q6NVY1	3-hydroxyisobutyryl-CoA hydrolase, mitochondrial	HIBCH	0.53	9.05E-04
Q9UBQ7	Glyoxylate reductase/hydroxypyruvate reductase	GRHPR	0.53	9.05E-04
A0A0A0MT83	Isovaleryl-CoA dehydrogenase, mitochondrial	IVD	0.52	9.05E-04
P08473	Neprilysin	MME	0.48	9.05E-04
O14745	Na(+)/H(+) exchange regulatory cofactor NHE-RF1	SLC9A3R1	0.46	9.05E-04

P05091	Aldehyde dehydrogenase, mitochondrial	ALDH2	0.45	9.05E-04
Q9NZN3	EH domain-containing protein 3	EHD3	0.33	9.05E-04
P30039	Phenazine biosynthesis-like domain-containing protein	PBLD	0.32	9.05E-04
P50440	Glycine amidinotransferase, mitochondrial	GATM	0.24	9.05E-04
Q9ULZ3	Apoptosis-associated speck-like protein containing a CARD	PYCARD	20.89	1.33E-03
J3QT28	Mitotic checkpoint protein BUB3 (Fragment)	BUB3	7.44	1.33E-03
P63220	40S ribosomal protein S21	RPS21	4.82	1.33E-03
A0A0U1RQV3	EGF-containing fibulin-like extracellular matrix protein 1 (Fragment)	EFEMP1	4.35	1.33E-03
P32455	Guanylate-binding protein 1	GBP1	3.81	1.33E-03
P05164	Myeloperoxidase	MPO	1.92	1.33E-03
P50552	Vasodilator-stimulated phosphoprotein	VASP	1.89	1.33E-03
O60500	Nephrin	NPHS1	0.64	1.33E-03
A0A0G2JIW1	Heat shock 70 kDa protein 1B	HSPA1B	0.63	1.33E-03
A0A087X0K9	Tight junction protein ZO-1	TJP1	0.62	1.33E-03
P30048	Thioredoxin-dependent peroxide reductase, mitochondrial	PRDX3	0.59	1.33E-03
P28331	NADH-ubiquinone oxidoreductase 75 kDa subunit, mitochondrial	NDUFS1	0.59	1.33E-03
P21399	Cytoplasmic aconitate hydratase	ACO1	0.52	1.33E-03
H3BNQ7	4-aminobutyrate aminotransferase, mitochondrial	ABAT	0.45	1.33E-03
Q16762	Thiosulfate sulfurtransferase	TST	0.44	1.33E-03
P42765	3-ketoacyl-CoA thiolase, mitochondrial	ACAA2	0.41	1.33E-03
Q08426	Peroxisomal bifunctional enzyme	EHHADH	0.40	1.33E-03
P00966	Argininosuccinate synthase	ASS1	0.31	1.33E-03
Q9NYU2	UDP-glucose:glycoprotein glucosyltransferase 1	UGGT1	7.68	1.76E-03
B0QYK4	EMI domain-containing protein 1	EMID1	5.17	1.76E-03
P04003	C4b-binding protein alpha chain	C4BPA	4.74	1.76E-03
H0Y5B4	60S ribosomal protein L36a	RPL36A	4.50	1.76E-03
B1ALD9	Periostin	POSTN	4.37	1.76E-03
P31146	Coronin-1A	CORO1A	3.46	1.76E-03
J3QSU6	Tenascin	TNC	3.10	1.76E-03
A0A0J9YY99	Uncharacterized protein (Fragment)		2.91	1.76E-03
P02751	Fibronectin	FN1	2.32	1.76E-03
Q9UJ70	N-acetyl-D-glucosamine kinase	NAGK	1.64	1.76E-03
Q9H444	Charged multivesicular body protein 4b	CHMP4B	1.62	1.76E-03
Q8NBS9	Thioredoxin domain-containing protein 5	TXNDC5	1.54	1.76E-03
Q96JY6	PDZ and LIM domain protein 2	PDLIM2	0.57	1.76E-03
Q07075	Glutamyl aminopeptidase	ENPEP	0.57	1.76E-03
Q96I99	Succinate--CoA ligase [GDP-forming] subunit beta, mitochondrial	SUCLG2	0.52	1.76E-03
P12277	Creatine kinase B-type	CKB	0.50	1.76E-03
P81605	Dermcidin	DCD	0.47	1.76E-03
P27144	Adenylate kinase 4, mitochondrial	AK4	0.43	1.76E-03
Q08257	Quinone oxidoreductase	CRYZ	0.43	1.76E-03
B7Z9I1	Medium-chain-specific acyl-CoA dehydrogenase, mitochondrial	ACADM	0.42	1.76E-03
P21695	Glycerol-3-phosphate dehydrogenase [NAD(+)], cytoplasmic	GPD1	0.38	1.76E-03
P50053	Ketohexokinase	KHK	0.30	1.76E-03
E5RI16	Protein FAM49B (Fragment)	FAM49B	14.07	2.38E-03
Q07954	Prolow-density lipoprotein receptor-related protein 1	LRP1	9.43	2.38E-03
E9PHS0	Glutathione S-transferase LANCL1 (Fragment)	LANCL1	3.31	2.38E-03
P62191	26S proteasome regulatory subunit 4	PSMC1	2.70	2.38E-03
Q9ULV4	Coronin-1C	CORO1C	2.25	2.38E-03

P52907	F-actin-capping protein subunit alpha-1	CAPZA1	2.19	2.38E-03
Q9Y3Z3	Deoxynucleoside triphosphate triphosphohydrolase SAMHD1	SAMHD1	2.07	2.38E-03
F2Z2Y8	Toll-interacting protein	TOLLIP	2.00	2.38E-03
A0A087X1Z3	Proteasome activator complex subunit 2	PSME2	1.73	2.38E-03
P62913	60S ribosomal protein L11	RPL11	1.54	2.38E-03
A0A3B3ITK7	Phosphoglucomutase-1	PGM1	0.65	2.38E-03
P11766	Alcohol dehydrogenase class-3	ADH5	0.62	2.38E-03
P30041	Peroxiredoxin-6	PRDX6	0.61	2.38E-03
A0A1B0GTG2	Alpha-aminoadipic semialdehyde dehydrogenase	ALDH7A1	0.58	2.38E-03
P05141	ADP/ATP translocase 2	SLC25A5	0.57	2.38E-03
Q3LXA3	Triokinase/FMN cyclase	TKFC	0.56	2.38E-03
P13716	Delta-aminolevulinic acid dehydratase	ALAD	0.50	2.38E-03
A0A0D9SFP2	Hydroxyacyl-coenzyme A dehydrogenase, mitochondrial	HADH	0.46	2.38E-03
P15144	Aminopeptidase N	ANPEP	0.45	2.38E-03
P04040	Catalase	CAT	0.44	2.38E-03
O95831	Apoptosis-inducing factor 1, mitochondrial	AIFM1	0.43	2.38E-03
P24752	Acetyl-CoA acetyltransferase, mitochondrial	ACAT1	0.39	2.38E-03
J3KS22	L-xylulose reductase (Fragment)	DCXR	0.36	2.38E-03
A0A3B3IS80	Fructose-bisphosphate aldolase	ALDOB	0.27	2.38E-03
A0A0S2Z4L3	Protein S isoform 2 (Fragment)	PROS1	23.23	3.18E-03
A0A075B788	Receptor-type tyrosine-protein phosphatase C	PTPRC	13.42	3.18E-03
H3BM42	Golgi apparatus protein 1, isoform CRA_c	GLG1	11.65	3.18E-03
A0A3B3IU24	Serine protease HTRA1	HTRA1	7.73	3.18E-03
P02675	Fibrinogen beta chain	FGB	4.68	3.18E-03
A0A0A0MSV6	Complement C1q subcomponent subunit B (Fragment)	C1QB	3.94	3.18E-03
C9JC84	Fibrinogen gamma chain	FGG	3.62	3.18E-03
Q92747	Actin-related protein 2/3 complex subunit 1A	ARPC1A	2.53	3.18E-03
P12814	Alpha-actinin-1	ACTN1	1.75	3.18E-03
H0Y8X4	2'-deoxynucleoside 5'-phosphate N-hydrolase 1 (Fragment)	DNPH1	0.63	3.18E-03
P49189	4-trimethylaminobutyraldehyde dehydrogenase	ALDH9A1	0.61	3.18E-03
O00592	Podocalyxin	PODXL	0.57	3.18E-03
P10809	60 kDa heat shock protein, mitochondrial	HSPD1	0.49	3.18E-03
C9JDE9	3-ketoacyl-CoA thiolase, peroxisomal	ACAA1	0.48	3.18E-03
H7C126	3-hydroxyisobutyryl-CoA hydrolase, mitochondrial (Fragment)	HIBCH	0.47	3.18E-03
A0A087WV24	Aromatic-L-amino-acid decarboxylase	DDC	0.46	3.18E-03
P68871	Hemoglobin subunit beta	HBB	0.44	3.18E-03
P05026	Sodium/potassium-transporting ATPase subunit beta-1	ATP1B1	0.44	3.18E-03
P09467	Fructose-1,6-bisphosphatase 1	FBP1	0.33	3.18E-03
Q68CK6	Acyl-coenzyme A synthetase ACSM2B, mitochondrial	ACSM2B	0.28	3.18E-03
Q9BVM4	Gamma-glutamylaminecyclotransferase	GGACT	0.18	3.18E-03
E9PLA9	Caprin-1 (Fragment)	CAPRIN1	4.74	4.49E-03
P08603	Complement factor H	CFH	3.51	4.49E-03
J3QSB5	60S ribosomal protein L36	RPL36	2.79	4.49E-03
D6RGG3	Collagen alpha-1(XII) chain	COL12A1	2.63	4.49E-03
Q92626	Peroxidasin homolog	PXDN	2.37	4.49E-03
P21980	Protein-glutamine gamma-glutamyltransferase 2	TGM2	2.33	4.49E-03
Q9UNZ2	NSFL1 cofactor p47	NSFL1C	2.14	4.49E-03
E5RJU9	Protein LYRIC	MTDH	1.52	4.49E-03
Q32Q12	Nucleoside diphosphate kinase	NME1-NME2	1.51	4.49E-03
O75874	Isocitrate dehydrogenase [NADP] cytoplasmic	IDH1	0.63	4.49E-03
P30046	D-dopachrome decarboxylase	DDT	0.62	4.49E-03
A0A087WT99	Ester hydrolase C11orf54	C11orf54	0.60	4.49E-03
P48735	Isocitrate dehydrogenase [NADP], mitochondrial	IDH2	0.53	4.49E-03
P02042	Hemoglobin subunit delta	HBD	0.44	4.49E-03

P34896	Serine hydroxymethyltransferase, cytosolic	SHMT1	0.42	4.49E-03
P45954	Short/branched chain specific acyl-CoA dehydrogenase, mitochondrial	ACADSB	0.23	4.49E-03
Q99536	Synaptic vesicle membrane protein VAT-1 homolog	VAT1	6.66	6.10E-03
A0A0B4J231	Immunoglobulin lambda-like polypeptide 5	IGLL5	4.05	6.10E-03
A0A087WX29	TAR DNA-binding protein 43 (Fragment)	TARDBP	4.02	6.10E-03
E9PQN9	Interferon-induced transmembrane protein 2	IFITM2	3.41	6.10E-03
P02671	Fibrinogen alpha chain	FGA	3.06	6.10E-03
P0DOY2	Immunoglobulin lambda constant 2	IGLC2	2.55	6.10E-03
O00303	Eukaryotic translation initiation factor 3 subunit F	EIF3F	2.14	6.10E-03
P28838	Cytosol aminopeptidase	LAP3	1.91	6.10E-03
J3KQL8	Apolipoprotein L2	APOL2	1.83	6.10E-03
P18085	ADP-ribosylation factor 4	ARF4	1.55	6.10E-03
P00740	Coagulation factor IX	F9	1.55	6.10E-03
P17174	Aspartate aminotransferase, cytoplasmic	GOT1	0.62	6.10E-03
Q9NPJ3	Acyl-coenzyme A thioesterase 13	ACOT13	0.59	6.10E-03
P32119	Peroxiredoxin-2	PRDX2	0.59	6.10E-03
O76041	Nebulette	NEBL	0.36	6.10E-03
P02730	Band 3 anion transport protein	SLC4A1	0.30	6.10E-03
Q9HCY8	Protein S100-A14	S100A14	19.49	8.06E-03
P27482	Calmodulin-like protein 3	CALML3	5.83	8.06E-03
Q9NR31	GTP-binding protein SAR1a	SAR1A	5.45	8.06E-03
P05198	Eukaryotic translation initiation factor 2 subunit 1	EIF2S1	4.96	8.06E-03
Q9Y6K5	2'-5'-oligoadenylate synthase 3	OAS3	4.02	8.06E-03
P42224	Signal transducer and activator of transcription 1-alpha/beta	STAT1	3.03	8.06E-03
B7ZKJ8	ITIH4 protein	ITIH4	2.97	8.06E-03
P12004	Proliferating cell nuclear antigen	PCNA	2.69	8.06E-03
A0A3B3ISR2	Complement C1r subcomponent	C1R	2.58	8.06E-03
H0Y2P0	CD44 antigen (Fragment)	CD44	2.19	8.06E-03
P04114	Apolipoprotein B-100	APOB	2.14	8.06E-03
P61769	Beta-2-microglobulin	B2M	1.99	8.06E-03
P15311	Ezrin	EZR	0.66	8.06E-03
P10768	S-formylglutathione hydrolase	ESD	0.64	8.06E-03
P00167	Cytochrome b5	CYB5A	0.63	8.06E-03
E7EQ64	Trypsin-1	PRSS1	0.63	8.06E-03
Q8TEW8	Partitioning defective 3 homolog B	PARD3B	0.60	8.06E-03
A0A087WY08	Thrombospondin type-1 domain-containing protein 7A	THSD7A	0.55	8.06E-03
H7C0J5	Centrosomal protein of 104 kDa	CEP104	0.48	8.06E-03
P00915	Carbonic anhydrase 1	CA1	0.46	8.06E-03
A8MW49	Fatty acid-binding protein, liver	FABP1	0.35	8.06E-03
Q9Y5K6	CD2-associated protein	CD2AP	11.78	1.05E-02
Q00688	Peptidyl-prolyl cis-trans isomerase FKBP3	FKBP3	4.55	1.05E-02
Q13185	Chromobox protein homolog 3	CBX3	4.39	1.05E-02
Q14651	Plastin-1	PLS1	3.15	1.05E-02
P29992	Guanine nucleotide-binding protein subunit alpha-11	GNA11	2.98	1.05E-02
P26447	Protein S100-A4	S100A4	2.52	1.05E-02
P02750	Leucine-rich alpha-2-glycoprotein	LRG1	2.48	1.05E-02
P14902	Indoleamine 2,3-dioxygenase 1	IDO1	2.29	1.05E-02
P55060	Exportin-2	CSE1L	2.21	1.05E-02
A0A0C4DFS8	Nicotinamide phosphoribosyltransferase	NAMPT	1.72	1.05E-02
P04004	Vitronectin	VTN	1.62	1.05E-02
O94760	N(G),N(G)-dimethylarginine dimethylaminohydrolase 1	DDAH1	0.64	1.05E-02
F8VW96	Cysteine and glycine-rich protein 2	CSRP2	0.56	1.05E-02
P20591	Interferon-induced GTP-binding protein Mx1	MX1	0.56	1.05E-02
P05154	Plasma serine protease inhibitor	SERPINA5	0.55	1.05E-02
A0A087WZY5	Collagen alpha-6(IV) chain	COL4A6	0.55	1.05E-02

P30084	Enoyl-CoA hydratase, mitochondrial	ECHS1	0.45	1.05E-02
A0A286YF22	D-3-phosphoglycerate dehydrogenase	PHGDH	0.43	1.05E-02
Q08431	Lactadherin	MFGE8	5.01	1.35E-02
P40121	Macrophage-capping protein	CAPG	4.79	1.35E-02
P43652	Afamin	AFM	4.17	1.35E-02
Q96DC9	Ubiquitin thioesterase OTUB2	OTUB2	3.86	1.35E-02
A6NF51	3'(2'),5'-bisphosphate nucleotidase 1	BPNT1	3.52	1.35E-02
A0A2R8Y5B3	Band 4,1-like protein 2 (Fragment)	EPB41L2	3.31	1.35E-02
O75556	Mammaglobin-B	SCGB2A1	3.22	1.35E-02
B5ME19	Eukaryotic translation initiation factor 3 subunit C-like protein	EIF3CL	2.93	1.35E-02
P51178	1-phosphatidylinositol 4,5-bisphosphate phosphodiesterase delta-1	PLCD1	2.34	1.35E-02
P01008	Antithrombin-III	SERPINC1	2.22	1.35E-02
Q9Y446	Plakophilin-3	PKP3	2.08	1.35E-02
P26373	60S ribosomal protein L13	RPL13	1.87	1.35E-02
P40306	Proteasome subunit beta type-10	PSMB10	1.77	1.35E-02
P05023	Sodium/potassium-transporting ATPase subunit alpha-1	ATP1A1	0.65	1.35E-02
Q15599	Na(+)/H(+) exchange regulatory cofactor NHE-RF2	SLC9A3R2	0.60	1.35E-02
A6NGU5	Putative glutathione hydrolase 3 proenzyme	GGT3P	0.58	1.35E-02
Q8N3V7	Synaptopodin	SYNPO	0.57	1.35E-02
P11277	Spectrin beta chain, erythrocytic	SPTB	0.56	1.35E-02
P82980	Retinol-binding protein 5	RBP5	0.48	1.35E-02
P02792	Ferritin light chain	FTL	0.35	1.35E-02
Q9BSE5	Agmatinase, mitochondrial	AGMAT	0.31	1.35E-02
P16444	Dipeptidase 1	DPEP1	0.23	1.35E-02
Q9UJY1	Heat shock protein beta-8	HSPB8	3.62	1.77E-02
E9PMV2	HLA class II histocompatibility antigen, DQ alpha 1 chain (Fragment)	HLA-DQA1	2.81	1.77E-02
P02748	Complement component C9	C9	1.83	1.77E-02
P18124	60S ribosomal protein L7	RPL7	1.82	1.77E-02
I3L0K7	Heat shock protein 75 kDa, mitochondrial	TRAP1	1.75	1.77E-02
G5E9W9	GTPase IMAP family member 4	GIMAP4	1.72	1.77E-02
P27338	Amine oxidase [flavin-containing] B	MAOB	0.61	1.77E-02
Q15274	Nicotinate-nucleotide pyrophosphorylase [carboxylating]	QPRT	0.59	1.77E-02
Q16698	2,4-dienoyl-CoA reductase, mitochondrial	DECR1	0.52	1.77E-02
A0A096LNH5	Glutamine amidotransferase-like class 1 domain-containing protein 3B, mitochondrial	GATD3B	0.37	1.77E-02
P02747	Complement C1q subcomponent subunit C	C1QC	10.77	2.24E-02
P62424	60S ribosomal protein L7a	RPL7A	6.31	2.24E-02
A0A286YEY1	Immunoglobulin heavy constant alpha 1 (Fragment)	IGHA1	4.30	2.24E-02
Q9UH99	SUN domain-containing protein 2	SUN2	3.90	2.24E-02
Q9H0U4	Ras-related protein Rab-1B	RAB1B	3.34	2.24E-02
Q14574	Desmocollin-3	DSC3	2.30	2.24E-02
B7Z5J4	Carboxypeptidase A4	CPA4	2.13	2.24E-02
Q13347	Eukaryotic translation initiation factor 3 subunit I	EIF3I	1.96	2.24E-02
A0A087WUM0	SYNJ2BP-COX16 readthrough (Fragment)	SYNJ2BP-COX16	1.85	2.24E-02
P50454	Serpin H1	SERPINH1	1.83	2.24E-02
H3BR90	Mesothelin (Fragment)	MSLN	1.79	2.24E-02
J3KTA4	Probable ATP-dependent RNA helicase DDX5	DDX5	1.74	2.24E-02
A0A087WXM6	60S ribosomal protein L17 (Fragment)	RPL17	1.64	2.24E-02
P35555	Fibrillin-1	FBN1	1.52	2.24E-02
A0A0D9SG72	Syntaxin-binding protein 1	STXBP1	0.62	2.24E-02
F5GZS6	4F2 cell-surface antigen heavy chain	SLC3A2	0.61	2.24E-02
Q9BW30	Tubulin polymerization-promoting protein family member 3	TPPP3	0.61	2.24E-02

Q9BPW8	Protein NipSnap homolog 1	NIPSNAP1	0.53	2.24E-02
P30043	Flavin reductase (NADPH)	BLVRB	0.48	2.24E-02
O14880	Microsomal glutathione S-transferase 3	MGST3	15.22	2.68E-02
E9PF63	Rho-associated protein kinase 2	ROCK2	12.40	2.68E-02
E5RJR5	S-phase kinase-associated protein 1	SKP1	4.05	2.68E-02
O75368	SH3 domain-binding glutamic acid-rich-like protein	SH3BGRL	4.00	2.68E-02
P08514	Integrin alpha-IIb	ITGA2B	3.42	2.68E-02
A0A0A0MSV9	Tapasin	TAPBP	2.80	2.68E-02
P19652	Alpha-1-acid glycoprotein 2	ORM2	2.71	2.68E-02
Q9Y6W5	Wiskott-Aldrich syndrome protein family member 2	WASF2	2.60	2.68E-02
G3V4U0	Fibulin-5	FBLN5	2.57	2.68E-02
P02743	Serum amyloid P-component	APCS	2.32	2.68E-02
A0A2Q2TTZ9	Immunoglobulin kappa variable 1-33	IGKV1D-33	2.23	2.68E-02
H7C0X5	Probable serine carboxypeptidase CPVL (Fragment)	CPVL	2.22	2.68E-02
A0A0G2JJZ9	Spliceosome RNA helicase DDX39B (Fragment)	DDX39B	1.99	2.68E-02
P13671	Complement component C6	C6	1.98	2.68E-02
A0A0A0MS08	Immunoglobulin heavy constant gamma 1 (Fragment)	IGHG1	1.92	2.68E-02
A0A075B716	40S ribosomal protein S17	RPS17	1.91	2.68E-02
P00488	Coagulation factor XIII A chain	F13A1	1.87	2.68E-02
P04275	von Willebrand factor	VWF	1.85	2.68E-02
M0R0F0	40S ribosomal protein S5 (Fragment)	RPS5	1.65	2.68E-02
K7EM38	Actin, cytoplasmic 2 (Fragment)	ACTG1	1.64	2.68E-02
F8WCA0	Vesicle-associated membrane protein 2	VAMP2	1.56	2.68E-02
P21291	Cysteine and glycine-rich protein 1	CSRP1	0.65	2.68E-02
P02452	Collagen alpha-1(I) chain	COL1A1	0.64	2.68E-02
P35914	Hydroxymethylglutaryl-CoA lyase, mitochondrial	HMGCL	0.63	2.68E-02
P02794	Ferritin heavy chain	FTH1	0.60	2.68E-02
Q9BTZ2	Dehydrogenase/reductase SDR family member 4	DHRS4	0.60	2.68E-02
Q00796	Sorbitol dehydrogenase	SORD	0.48	2.68E-02
P05413	Fatty acid-binding protein, heart	FABP3	0.39	2.68E-02
P01624	Immunoglobulin kappa variable 3-15	IGKV3-15	4.97	3.35E-02
E9PKH6	NADH dehydrogenase [ubiquinone] iron-sulfur protein 8, mitochondrial (Fragment)	NDUFS8	4.37	3.35E-02
A0A096LNZ9	Ubiquitin-like protein ISG15 (Fragment)	ISG15	3.78	3.35E-02
P69892	Hemoglobin subunit gamma-2	HBG2	3.51	3.35E-02
P07360	Complement component C8 gamma chain	C8G	2.30	3.35E-02
P26640	Valine--tRNA ligase	VARS	2.00	3.35E-02
Q9GZM7	Tubulointerstitial nephritis antigen-like	TINAGL1	1.97	3.35E-02
Q9NZM1	Myoferlin	MYOF	1.78	3.35E-02
O95147	Dual specificity protein phosphatase 14	DUSP14	1.65	3.35E-02
A0A0D9SF53	ATP-dependent RNA helicase DDX3X	DDX3X	1.61	3.35E-02
P16949	Stathmin	STMN1	1.61	3.35E-02
Q06323	Proteasome activator complex subunit 1	PSME1	1.58	3.35E-02
A2ACR1	Proteasome subunit beta	PSMB9	1.56	3.35E-02
P09488	Glutathione S-transferase Mu 1	GSTM1	0.58	3.35E-02
P27487	Dipeptidyl peptidase 4	DPP4	0.55	3.35E-02
Q6UWW0	Lipocalin-15	LCN15	12.76	4.10E-02
P05534	HLA class I histocompatibility antigen, A-24 alpha chain	HLA-A	8.28	4.10E-02
Q9Y624	Junctional adhesion molecule A	F11R	5.16	4.10E-02
Q10589	Bone marrow stromal antigen 2	BST2	3.08	4.10E-02
A0A0B4J2H0	Immunoglobulin heavy variable 1-69D	IGHV1-69D	2.83	4.10E-02
I3L4J1	Uncharacterized protein (Fragment), serait la Vesicle-fusing ATPase	NSF	2.07	4.10E-02
E9PBF6	Lamin-B1	LMNB1	2.05	4.10E-02

P00747	Plasminogen	PLG	1.80	4.10E-02
P17661	Desmin	DES	1.80	4.10E-02
P23142	Fibulin-1	FBLN1	1.79	4.10E-02
Q15582	Transforming growth factor-beta-induced protein ig-h3	TGFBI	1.68	4.10E-02
A0A0G2JJQ8	HLA class II histocompatibility antigen, DR beta 4 chain	HLA-DRB4	1.56	4.10E-02
B9A067	MICOS complex subunit MIC60	IMMT	0.65	4.10E-02
Q5IJ48	Protein crumbs homolog 2	CRB2	0.59	4.10E-02
Q9Y617	Phosphoserine aminotransferase	PSAT1	0.57	4.10E-02
P12273	Prolactin-inducible protein	PIP	0.55	4.10E-02
Q86YZ3	Hornerin	HRNR	0.37	4.10E-02
Q08AH3	Acyl-coenzyme A synthetase ACSM2A, mitochondrial	ACSM2A	0.21	4.10E-02
Q3ZCM7	Tubulin beta-8 chain	TUBB8	4.26	4.99E-02
A0A1B0GUU9	Immunoglobulin heavy constant mu (Fragment)	IGHM	3.99	4.99E-02
Q9NP72	Ras-related protein Rab-18	RAB18	3.67	4.99E-02
F8VY02	Endoplasmic reticulum resident protein 29	ERP29	3.33	4.99E-02
O00391	Sulfhydryl oxidase 1	QSOX1	2.89	4.99E-02
A0A2R8Y7G9	Uncharacterized protein	H3Y1	2.68	4.99E-02
A0A087X232	Complement C1s subcomponent	C1S	2.68	4.99E-02
P80188	Neutrophil gelatinase-associated lipocalin	LCN2	2.66	4.99E-02
P01834	Immunoglobulin kappa constant	IGKC	2.38	4.99E-02
Q92973	Transportin-1	TNPO1	2.12	4.99E-02
Q6UVK1	Chondroitin sulfate proteoglycan 4	CSPG4	1.81	4.99E-02
A0A087WUQ6	Glutathione peroxidase	GPX1	1.79	4.99E-02
P60842	Eukaryotic initiation factor 4A-I	EIF4A1	1.74	4.99E-02
P05155	Plasma protease C1 inhibitor	SERPING1	1.54	4.99E-02
P62136	Serine/threonine-protein phosphatase PP1-alpha catalytic subunit	PPP1CA	1.53	4.99E-02
E9PF16	Acyl-CoA synthetase family member 2, mitochondrial	ACSF2	0.54	4.99E-02
F8VY04	Adenylate kinase 2, mitochondrial	AK2	0.51	4.99E-02
Q8NE62	Choline dehydrogenase, mitochondrial	CHDH	0.44	4.99E-02

Non-parametric Mann-Whitney tests were performed to compare the protein expressions between the chronic active antibody-mediated rejection (caABMR) and stable graft (SG) group. P-values were secondarily adjusted according to the Benjamini-Hochberg correction. Of note, 4 proteins were removed from this list (KRT9, KRT7, KRT87P and KRTAP13-2), being a contaminant from the epidermis. One protein appear with two UniProt entries: HIBCH. Abbreviations: caABMR, active antibody-mediated rejection; SG, stable graft control.

Supplemental Table S3. Detailed histological scores and relative protein abundances of the five antibodies tested by immunohistochemistry for each analyzed case

		WARS1		TYMP		GBP1		CORO1A		EFHD2	
Group	Case number	Histological score	Normalized relative protein abundance	Histological score	Normalized relative protein abundance	Histological score	Normalized relative protein abundance	Histological score	Normalized relative protein abundance	Histological score	Normalized relative protein abundance
SG	1/2			0.00	7.44E+05	0.25	1.07E+06	3.50	4.69E+05		
SG	3/4	0.38	4.80E+06	0.29	4.37E+05	0.15	1.26E+06	3.24	4.37E+05	1.50	1.71E+05
SG	9/10	0.50	4.17E+06	0.14	6.40E+05						
SG	11/12	2.00	5.39E+06	1.36	1.17E+06	0.32	1.77E+06	7.27	6.56E+05	3.42	1.73E+05
SG	13/14	0.50	3.98E+06	0.44	8.03E+05	0.06	1.62E+06	7.75	8.01E+05		
SG	15/16	0.00	4.01E+06	0.13	4.57E+05	0.00	9.94E+05	3.00	7.29E+05	1.40	5.18E+04
SG	17/18	0.50	4.15E+06	0.50	7.32E+05						
SG	19/20	0.50	4.85E+06	0.27	1.13E+06	0.00	1.03E+06	2.36	5.62E+05	0.56	2.14E+05
aABMR	21/22	1.14	1.22E+07								
aABMR	23/24	3.00	1.86E+07	3.00	8.27E+06	3.00	7.69E+06				
aABMR	25/26	0.40	6.97E+06	0.56	1.89E+06	0.13	1.79E+06	8.17	1.15E+06	6.46	8.53E+05
aABMR	27/28			1.67	2.59E+06			15.00	1.76E+06	9.85	6.54E+05
aABMR	29/30										
aABMR	33/34	1.60	9.40E+06	2.08	3.40E+06	3.00	7.00E+06				
aABMR	37/38	3.00	4.00E+07	2.00	1.78E+07	3.00	3.08E+07				
caABMR	41/42	2.60	1.68E+07	2.75	6.19E+06	2.63	9.53E+06	19.22	2.72E+06	12.83	9.66E+05
caABMR	43/44	2.37	1.05E+07	2.20	3.22E+06	0.75	3.66E+06	19.58	2.14E+06	9.44	8.94E+05
caABMR	45/46	1.47	1.05E+07	1.84	2.96E+06	1.81	5.93E+06	13.76	2.02E+06	9.87	8.18E+05
caABMR	47/48	1.22	7.59E+06	1.82	1.88E+06			11.50	2.83E+06	7.71	8.60E+05
caABMR	49/50	1.25	5.46E+06	0.62	1.43E+06	0.00	1.54E+06	9.05	6.47E+05	5.00	3.89E+05
caABMR	53/54	1.50	1.17E+07	2.05	4.33E+06	1.38	3.58E+06	18.44	2.16E+06	8.76	6.06E+05
caABMR	55/56	2.67	1.09E+07	2.05	3.07E+06	2.25	5.19E+06	16.44	1.87E+06	6.75	6.88E+05
caABMR	57/58	0.78	9.28E+06	0.68	3.08E+06	0.40	1.94E+06			7.92	4.40E+05
caABMR	61/62										
caABMR	63/64	1.40	1.41E+07	2.54	4.47E+06	0.27	3.42E+06			6.93	9.98E+05

Normalized relative protein abundance is presented as acquired by mass spectrometry analysis. A histological glomerular score was visually assessed for each slide and each antibody by a pathologist (BC) for a semi-quantitative assessment of the *in situ* expression. For WARS1, GBP1 and TYMP antibodies, scores were calculated for each case by assessing the mean intensity of staining of the glomeruli, by visually evaluating the number of positive glomeruli and their respective intensity of staining (0 to 3+). Only cases with a minimum of 4 non-sclerotic glomeruli were considered for this semi-quantitative evaluation, where most cases had a diffuse pattern of staining (a majority of glomeruli showed positivity). For the CORO1A and EFHD2 antibodies, scores were assessed by calculating the mean number of positive cells *per* glomerular section with a visual enumeration. Only cases with at least 8 non-sclerotic glomeruli were assessed for this quantitative enumeration, as glomerulitis is often a focal lesion. Hence non available data correspond to cases with insufficient remaining material for staining assessment. Case number refers to each replicate number. Abbreviations: aABMR, active antibody-mediated rejection; caABMR, chronic active antibody-mediated rejection; SG, stable graft control.

Supplemental Table S4. List of the 137 proteins differentiating chronic active from active antibody-mediated glomerular injuries, in ascending order of adjusted p-values

UniProt access	Protein name	Corresponding gene name	Fold-change caABMR/aABMR	Adjusted p-value
P08603	Complement factor H	CFH	2.83	3.80E-03
A0A3B3IU24	Serine protease HTRA1	HTRA1	7.56	6.36E-03
A0A286YEY1	Immunoglobulin heavy constant alpha 1 (Fragment)	IGHA1	3.29	6.36E-03
A0A0S2Z4L3	Protein S isoform 2 (Fragment)	PROS1	13.80	6.36E-03
A0A087X0K0	Collagen alpha-1(XV) chain	COL15A1	3.89	6.36E-03
P55884	Eukaryotic translation initiation factor 3 subunit B	EIF3B	2.61	6.36E-03
Q08431	Lactadherin	MFGE8	2.00	6.36E-03
O60506	Heterogeneous nuclear ribonucleoprotein Q	SYNCRIP	1.77	6.36E-03
B1ALD9	Periostin	POSTN	3.84	6.36E-03
Q6PCB0	von Willebrand factor A domain-containing protein 1	VWA1	2.55	6.36E-03
P05141	ADP/ATP translocase 2	SLC25A5	0.60	6.36E-03
P02652	Apolipoprotein A-II	APOA2	2.23	6.36E-03
B7ZKJ8	ITIH4 protein	ITIH4	2.60	6.36E-03
P01008	Antithrombin-III	SERPINC1	2.12	6.36E-03
Q9BXR6	Complement factor H-related protein 5	CFHR5	2.70	6.36E-03
P21589	5'-nucleotidase	NT5E	2.84	6.36E-03
P00747	Plasminogen	PLG	1.84	6.36E-03
P14550	Aldo-keto reductase family 1 member A1	AKR1A1	0.65	6.36E-03
Q9UH99	SUN domain-containing protein 2	SUN2	7.34	6.36E-03
A0A0J9YY99	Uncharacterized protein (Fragment)		9.16	8.38E-03
A0A3B3ISR2	Complement C1r subcomponent	C1R	2.67	8.38E-03
O75368	SH3 domain-binding glutamic acid-rich-like protein	SH3BGRL	6.17	9.78E-03
P01624	Immunoglobulin kappa variable 3-15	IGKV3-15	4.29	9.78E-03
P0DOY2	Immunoglobulin lambda constant 2	IGLC2	2.46	9.78E-03
P05155	Plasma protease C1 inhibitor	SERPING1	1.75	9.78E-03
P02743	Serum amyloid P-component	APCS	1.89	9.78E-03
Q13347	Eukaryotic translation initiation factor 3 subunit I	EIF3I	1.75	1.19E-02
P04003	C4b-binding protein alpha chain	C4BPA	3.66	1.19E-02
Q14112	Nidogen-2	NID2	2.17	1.19E-02
P01037	Cystatin-SN	CST1	1.65	1.19E-02
A0A087X232	Complement C1s subcomponent	C1S	2.73	1.54E-02
Q99536	Synaptic vesicle membrane protein VAT-1 homolog	VAT1	4.56	1.54E-02
Q9Y2S2	Lambda-crystallin homolog	CRYL1	0.55	1.68E-02
Q93088	Betaine--homocysteine S-methyltransferase 1	BHMT	0.52	1.68E-02
P27169	Serum paraoxonase/arylesterase 1	PON1	3.18	1.68E-02
C9JC84	Fibrinogen gamma chain	FGG	2.69	1.68E-02
P02671	Fibrinogen alpha chain	FGA	2.57	1.68E-02
P55060	Exportin-2	CSE1L	2.22	1.68E-02
D6RGG3	Collagen alpha-1(XII) chain	COL12A1	1.93	1.68E-02
P02748	Complement component C9	C9	1.83	1.68E-02
A0A1B0GU86	Aminoacylase-1	ACY1	0.42	1.93E-02
P33176	Kinesin-1 heavy chain	KIF5B	1.78	1.93E-02
P02675	Fibrinogen beta chain	FGB	3.16	1.93E-02
Q9GZM7	Tubulointerstitial nephritis antigen-like	TINAGL1	2.30	1.93E-02
H3BNQ7	4-aminobutyrate aminotransferase, mitochondrial	ABAT	0.58	1.93E-02
A0A0A0MS41	Sideroflexin	SFXN3	1.72	1.93E-02
P43652	Afamin	AFM	3.23	1.93E-02
P00966	Argininosuccinate synthase	ASS1	0.49	2.22E-02
P00918	Carbonic anhydrase 2	CA2	0.54	2.22E-02

P21695	Glycerol-3-phosphate dehydrogenase [NAD(+)], cytoplasmic	GPD1	0.41	2.22E-02
P02787	Serotransferrin	TF	1.76	2.22E-02
P01023	Alpha-2-macroglobulin	A2M	2.12	2.22E-02
O14879	Interferon-induced protein with tetratricopeptide repeats 3	IFIT3	1.94	2.22E-02
P00740	Coagulation factor IX	F9	1.61	2.22E-02
Q16762	Thiosulfate sulfurtransferase	TST	0.49	2.39E-02
A0A087WUM0	SYNJ2BP-COX16 readthrough (Fragment)	SYNJ2BP-COX16	2.18	2.39E-02
A0A3B3IS80	Fructose-bisphosphate aldolase	ALDOB	0.37	2.39E-02
P09210	Glutathione S-transferase A2	GSTA2	0.37	2.39E-02
P16444	Dipeptidase 1	DPEP1	0.28	2.39E-02
P02747	Complement C1q subcomponent subunit C	C1QC	8.87	2.39E-02
Q14574	Desmocollin-3	DSC3	2.92	2.39E-02
A0A0B4J231	Immunoglobulin lambda-like polypeptide 5	IGLL5	2.44	2.39E-02
E9PQN9	Interferon-induced transmembrane protein 2	IFITM2	1.99	2.39E-02
Q3ZCM7	Tubulin beta-8 chain	TUBB8	3.85	2.39E-02
Q96KP4	Cytosolic non-specific dipeptidase	CNDP2	0.61	2.39E-02
P35555	Fibrillin-1	FBN1	1.79	2.39E-02
Q96CX2	BTB/POZ domain-containing protein KCTD12	KCTD12	1.78	2.84E-02
P23142	Fibulin-1	FBLN1	1.76	2.84E-02
P30038	Delta-1-pyrroline-5-carboxylate dehydrogenase, mitochondrial	ALDH4A1	0.42	2.84E-02
Q9BSE5	Agmatinase, mitochondrial	AGMAT	0.32	2.84E-02
P27482	Calmodulin-like protein 3	CALML3	3.79	2.84E-02
H0YJW3	Alpha-actinin-1 (Fragment)	ACTN1	1.77	2.84E-02
P0C0L4	Complement C4-A	C4A	1.59	3.03E-02
P09467	Fructose-1,6-bisphosphatase 1	FBP1	0.40	3.03E-02
P16949	Stathmin	STMN1	1.89	3.03E-02
C9JDE9	3-ketoacyl-CoA thiolase, peroxisomal	ACAA1	0.65	3.03E-02
E5RFU2	Bifunctional epoxide hydrolase 2	EPHX2	0.52	3.03E-02
B1AHL2	Fibulin-1	FBLN1	1.85	3.03E-02
Q07954	Prolow-density lipoprotein receptor-related protein 1	LRP1	6.67	3.03E-02
Q9H0U4	Ras-related protein Rab-1B	RAB1B	3.50	3.03E-02
Q16822	Phosphoenolpyruvate carboxykinase [GTP], mitochondrial	PCK2	0.41	3.03E-02
P45954	Short/branched chain specific acyl-CoA dehydrogenase, mitochondrial	ACADSB	0.64	3.03E-02
O00303	Eukaryotic translation initiation factor 3 subunit F	EIF3F	1.60	3.03E-02
A0A075B730	Epiplakin	EPPK1	1.94	3.03E-02
B0QYK4	EMI domain-containing protein 1	EMID1	2.24	3.03E-02
P29992	Guanine nucleotide-binding protein subunit alpha-11	GNA11	3.31	3.03E-02
P98160	Basement membrane-specific heparan sulfate proteoglycan core protein	HSPG2	1.79	3.03E-02
E7ENL6	Collagen alpha-3(VI) chain	COL6A3	1.59	3.13E-02
P10809	60 kDa heat shock protein, mitochondrial	HSPD1	0.51	3.13E-02
A0A0D9SFP2	Hydroxyacyl-coenzyme A dehydrogenase, mitochondrial	HADH	0.58	3.13E-02
P80365	Corticosteroid 11-beta-dehydrogenase isozyme 2	HSD11B2	0.20	3.13E-02
P30039	Phenazine biosynthesis-like domain-containing protein	PBLD	0.42	3.13E-02
A0A0A0MSV6	Complement C1q subcomponent subunit B (Fragment)	C1QB	2.66	3.13E-02
A0A286YES1	Immunoglobulin heavy constant gamma 3 (Fragment)	IGHG3	1.93	3.13E-02
Q8WVV4	Protein POF1B	POF1B	1.90	3.13E-02
H0Y4R1	Inosine-5'-monophosphate dehydrogenase 2 (Fragment)	IMPDH2	2.02	3.13E-02

P01834	Immunoglobulin kappa constant	IGKC	2.64	3.13E-02
A0A0D9SF53	ATP-dependent RNA helicase DDX3X	DDX3X	1.55	3.13E-02
P02751	Fibronectin	FN1	1.86	3.13E-02
P41091	Eukaryotic translation initiation factor 2 subunit 3	EIF2S3	3.22	3.13E-02
P50440	Glycine amidinotransferase, mitochondrial	GATM	0.51	3.13E-02
P42765	3-ketoacyl-CoA thiolase, mitochondrial	ACAA2	0.42	3.13E-02
Q02252	Methylmalonate-semialdehyde dehydrogenase [acylating], mitochondrial	ALDH6A1	0.35	3.13E-02
Q6IB77	Glycine N-acyltransferase	GLYAT	0.48	3.13E-02
P04004	Vitronectin	VTN	1.53	3.13E-02
E9PFZ2	Ceruloplasmin	CP	1.63	3.13E-02
P08514	Integrin alpha-IIb	ITGA2B	3.00	3.13E-02
J3QSU6	Tenascin	TNC	1.97	3.55E-02
A0A087WX29	TAR DNA-binding protein 43 (Fragment)	TARDBP	1.67	3.55E-02
E5RI16	Protein FAM49B (Fragment)	FAM49B	8.66	3.55E-02
P36952	Serpin B5	SERPINB5	2.49	3.55E-02
Q6ZVX7	F-box only protein 50	NCCRP1	2.64	3.55E-02
Q6NVY1	3-hydroxyisobutyryl-CoA hydrolase, mitochondrial	HIBCH	0.62	3.55E-02
G3XAI2	Laminin subunit beta-1	LAMB1	1.64	3.55E-02
P01031	Complement C5	C5	2.39	3.55E-02
H7C126	3-hydroxyisobutyryl-CoA hydrolase, mitochondrial (Fragment)	HIBCH	0.51	3.55E-02
P07195	L-lactate dehydrogenase B chain	LDHB	0.63	3.55E-02
P07360	Complement component C8 gamma chain	C8G	2.46	3.55E-02
Q9HCY8	Protein S100-A14	S100A14	11.24	3.55E-02
Q96DG6	Carboxymethylenebutenolidase homolog	CMBL	0.52	4.03E-02
P07858	Cathepsin B	CTSB	4.20	4.03E-02
H3BPK3	Hydroxyacylglutathione hydrolase, mitochondrial (Fragment)	HAGH	0.61	4.03E-02
A0A0C4DH41	Immunoglobulin heavy variable 4-61	IGHV4-61	2.01	4.03E-02
A0A0A0MS08	Immunoglobulin heavy constant gamma 1 (Fragment)	IGHG1	1.69	4.03E-02
P04114	Apolipoprotein B-100	APOB	2.09	4.03E-02
P02749	Beta-2-glycoprotein 1	APOH	2.67	4.03E-02
A0A2Q2TTZ9	Immunoglobulin kappa variable 1-33	IGKV1D-33	1.57	4.03E-02
Q8NE62	Choline dehydrogenase, mitochondrial	CHDH	0.64	4.03E-02
P12814	Alpha-actinin-1	ACTN1	1.53	4.03E-02
F8VY02	Endoplasmic reticulum resident protein 29	ERP29	2.08	4.03E-02
P02649	Apolipoprotein E	APOE	2.34	4.03E-02
P50053	Ketohexokinase	KHK	0.39	4.67E-02
P43121	Cell surface glycoprotein MUC18	MCAM	1.61	4.67E-02
P38117	Electron transfer flavoprotein subunit beta	ETFB	0.64	4.67E-02
A0A0G2JMX7	Microtubule-associated protein	MAPT	2.02	4.67E-02
P30048	Thioredoxin-dependent peroxide reductase, mitochondrial	PRDX3	0.53	4.67E-02
P07954	Fumarate hydratase, mitochondrial	FH	0.57	4.67E-02
P00734	Prothrombin	F2	2.16	4.67E-02
P48147	Prolyl endopeptidase	PREP	1.83	4.67E-02
E9PLA9	Caprin-1 (Fragment)	CAPRIN1	2.70	4.67E-02

Non-parametric Mann-Whitney tests were performed to compare the protein expressions between the chronic active antibody-mediated rejection (caABMR) and active ABMR (aABMR) group. P-values were secondarily adjusted according to the Benjamini-Hochberg correction. Of note, one protein was removed from this list (KRT19), being a contaminant from the epidermis. Three proteins appear with two UniProt entries: ACTN1, FBLN1 and HIBCH. Abbreviations: caABMR, active antibody-mediated rejection; SG, stable graft control.

Supplemental Table S5. List of the 135 extracellular matrix proteins of this study according to the Matrisome Project database

Division	Category	Protein name	Corresponding gene name	UniProt access
Core matrisome	Collagens	Collagen alpha-1(XII) chain	COL12A1	D6RGG3
Core matrisome	Collagens	Collagen alpha-1(XIV) chain	COL14A1	J3QT83
Core matrisome	Collagens	Collagen alpha-1(XVIII) chain	COL18A1	P39060
Core matrisome	Collagens	Collagen alpha-1(I) chain	COL1A1	P02452
Core matrisome	Collagens	Collagen alpha-2(I) chain	COL1A2	A0A087WTA8
Core matrisome	Collagens	Collagen alpha-1(IV) chain	COL4A1	P02462
Core matrisome	Collagens	Collagen alpha-2(IV) chain	COL4A2	P08572
Core matrisome	Collagens	Collagen alpha-3(IV) chain	COL4A3	Q01955
Core matrisome	Collagens	Collagen alpha-4(IV) chain	COL4A4	P53420
Core matrisome	Collagens	Collagen alpha-5(IV) chain	COL4A5	P29400
Core matrisome	Collagens	Collagen alpha-6(IV) chain	COL4A6	A0A087WZY5
Core matrisome	Collagens	Collagen alpha-1(VI) chain	COL6A1	A0A087X0S5
Core matrisome	Collagens	Collagen alpha-2(VI) chain	COL6A2	P12110
Core matrisome	Collagens	Collagen alpha-3(VI) chain	COL6A3	E7ENL6
Core matrisome	Collagens	Collagen alpha-3(VI) chain	COL6A3	P12111
Core matrisome	Collagens	Collagen alpha-1(VII) chain	COL7A1	Q02388
Core matrisome	Collagens	Collagen alpha-1(XV) chain	COL15A1	A0A087X0K0
Core matrisome	ECM Glycoproteins	Agrin	AGRN	O00468
Core matrisome	ECM Glycoproteins	Deleted in malignant brain tumors 1 protein	DMBT1	Q9UGM3
Core matrisome	ECM Glycoproteins	Extracellular matrix protein 1	ECM1	Q16610
Core matrisome	ECM Glycoproteins	EGF-containing fibulin-like extracellular matrix protein 1	EFEMP1	A0A0U1RQV3
Core matrisome	ECM Glycoproteins	EMI domain-containing protein 1	EMID1	B0QYK4
Core matrisome	ECM Glycoproteins	EMILIN-1	EMILIN1	Q9Y6C2
Core matrisome	ECM Glycoproteins	Fibulin-1	FBLN1	B1AHL2
Core matrisome	ECM Glycoproteins	Fibulin-1	FBLN1	P23142
Core matrisome	ECM Glycoproteins	Fibulin-5	FBLN5	G3V4U0
Core matrisome	ECM Glycoproteins	Fibrillin-1	FBN1	P35555
Core matrisome	ECM Glycoproteins	Fibrinogen alpha chain	FGA	P02671
Core matrisome	ECM Glycoproteins	Fibrinogen beta chain	FGB	P02675
Core matrisome	ECM Glycoproteins	Fibrinogen gamma chain	FGG	C9JC84
Core matrisome	ECM Glycoproteins	Fibronectin	FN1	P02751
Core matrisome	ECM Glycoproteins	Laminin subunit alpha-2	LAMA2	A0A087WX80
Core matrisome	ECM Glycoproteins	Laminin subunit alpha-4	LAMA4	A0A0A0MQS9
Core matrisome	ECM Glycoproteins	Laminin subunit alpha-5	LAMA5	O15230
Core matrisome	ECM Glycoproteins	Laminin subunit beta-1	LAMB1	G3XAI2
Core matrisome	ECM Glycoproteins	Laminin subunit beta-2	LAMB2	P55268
Core matrisome	ECM Glycoproteins	Laminin subunit gamma-1	LAMC1	P11047
Core matrisome	ECM Glycoproteins	Leucine-rich alpha-2-glycoprotein	LRG1	P02750
Core matrisome	ECM Glycoproteins	Matrix Gla protein	MGP	P08493
Core matrisome	ECM Glycoproteins	Multimerin-2	MMRN2	Q9H8L6
Core matrisome	ECM Glycoproteins	Nidogen-1	NID1	P14543
Core matrisome	ECM Glycoproteins	Periostin	POSTN	B1ALD9
Core matrisome	ECM Glycoproteins	Nephronectin	NPNT	D6RH31
Core matrisome	ECM Glycoproteins	Papilin	PAPLN	O95428
Core matrisome	ECM Glycoproteins	Peroxidasin homolog	PXDN	Q92626
Core matrisome	ECM Glycoproteins	Transforming growth factor-beta-induced protein ig-h3	TGFBI	Q15582
Core matrisome	ECM Glycoproteins	Thrombospondin-1	THBS1	P07996
Core matrisome	ECM Glycoproteins	Tubulointerstitial nephritis antigen	TINAG	Q9UJW2
Core matrisome	ECM Glycoproteins	Tubulointerstitial nephritis antigen-like	TINAGL1	Q9GZM7
Core matrisome	ECM Glycoproteins	Tenascin	TNC	J3QSU6
Core matrisome	ECM Glycoproteins	Tenascin-X	TNXB	A0A140T8Y3
Core matrisome	ECM Glycoproteins	Vitronectin	VTN	P04004
Core matrisome	ECM Glycoproteins	von Willebrand factor A domain-containing protein 1	VWA1	Q6PCB0
Core matrisome	ECM Glycoproteins	von Willebrand factor	VWF	P04275
Core matrisome	ECM Glycoproteins	Nidogen-2	NID2	Q14112
Core matrisome	ECM Glycoproteins	Lactadherin	MFGE8	Q08431
Core matrisome	Proteoglycans	Biglycan	BGN	P21810
Core matrisome	Proteoglycans	Decorin	DCN	P07585
Core matrisome	Proteoglycans	Basement membrane-specific heparan sulfate proteoglycan core protein	HSPG2	P98160
Core matrisome	Proteoglycans	Lumican	LUM	P51884

Core matrisome	Proteoglycans	Mimecan	OGN	P20774
Core matrisome	Proteoglycans	Versican core protein	VCAN	E9PF17
Matrisome-associated	ECM Regulators	Serine protease HTRA1	HTRA1	A0A3B3IU24
Matrisome-associated	ECM Regulators	ITIH4 protein	ITIH4	B7ZKJ8
Matrisome-associated	ECM Regulators	Alpha-2-macroglobulin	A2M	P01023
Matrisome-associated	ECM Regulators	Disintegrin and metalloproteinase domain-containing protein 10	ADAM10	O14672
Matrisome-associated	ECM Regulators	Angiotensinogen	AGT	P01019
Matrisome-associated	ECM Regulators	Protein AMBP	AMBP	P02760
Matrisome-associated	ECM Regulators	Cystatin-M	CST6	Q15828
Matrisome-associated	ECM Regulators	Cystatin-A	CSTA	P01040
Matrisome-associated	ECM Regulators	Cystatin-B	CSTB	P04080
Matrisome-associated	ECM Regulators	Cathepsin B	CTSB	P07858
Matrisome-associated	ECM Regulators	Cathepsin D	CTSD	A0A1B0GVD5
Matrisome-associated	ECM Regulators	Cathepsin G	CTSG	P08311
Matrisome-associated	ECM Regulators	Cathepsin Z	CTSZ	Q9UBR2
Matrisome-associated	ECM Regulators	Coagulation factor XIII A chain	F13A1	P00488
Matrisome-associated	ECM Regulators	Prothrombin	F2	P00734
Matrisome-associated	ECM Regulators	Coagulation factor IX	F9	P00740
Matrisome-associated	ECM Regulators	Histidine-rich glycoprotein	HRG	P04196
Matrisome-associated	ECM Regulators	Antithrombin-III	SERPINC1	P01008
Matrisome-associated	ECM Regulators	Inter-alpha-trypsin inhibitor heavy chain H1	ITIH1	P19827
Matrisome-associated	ECM Regulators	Inter-alpha-trypsin inhibitor heavy chain H2	ITIH2	P19823
Matrisome-associated	ECM Regulators	Inter-alpha-trypsin inhibitor heavy chain H5	ITIH5	C9J2H1
Matrisome-associated	ECM Regulators	Kininogen-1	KNG1	P01042
Matrisome-associated	ECM Regulators	Apolipoprotein(a)	LPA	A0A087WWY0
Matrisome-associated	ECM Regulators	Plasminogen	PLG	P00747
Matrisome-associated	ECM Regulators	Trypsin-1	PRSS1	E7EQ64
Matrisome-associated	ECM Regulators	Trypsin-3	PRSS3	B1AN99
Matrisome-associated	ECM Regulators	Serpin A12	SERPINA12	Q8IW75
Matrisome-associated	ECM Regulators	Alpha-1-antichymotrypsin	SERPINA3	P01011
Matrisome-associated	ECM Regulators	Plasma serine protease inhibitor	SERPINA5	P05154
Matrisome-associated	ECM Regulators	Plasma protease C1 inhibitor	SERPING1	P05155
Matrisome-associated	ECM Regulators	Serpin B12	SERPINB12	Q96P63
Matrisome-associated	ECM Regulators	Serpin B3	SERPINB3	P29508
Matrisome-associated	ECM Regulators	Serpin B4	SERPINB4	H0Y5H9
Matrisome-associated	ECM Regulators	Serpin B5	SERPINB5	P36952
Matrisome-associated	ECM Regulators	Serpin B6	SERPINB6	A0A024QZX5
Matrisome-associated	ECM Regulators	Serpin B9	SERPINB9	P50453
Matrisome-associated	ECM Regulators	Cystatin-SN	CST1	P01037
Matrisome-associated	ECM Regulators	Heparin cofactor 2	SERPIND1	P05546
Matrisome-associated	ECM Regulators	Pigment epithelium-derived factor	SERPINF1	P36955
Matrisome-associated	ECM Regulators	Serpin H1	SERPINH1	P50454
Matrisome-associated	ECM Regulators	Antileukoproteinase	SLPI	P03973
Matrisome-associated	ECM Regulators	Protein-glutamine gamma-glutamyltransferase K	TGM1	P22735
Matrisome-associated	ECM Regulators	Protein-glutamine gamma-glutamyltransferase 2	TGM2	P21980
Matrisome-associated	ECM Regulators	Protein-glutamine gamma-glutamyltransferase E	TGM3	Q08188
Matrisome-associated	ECM Regulators	Leukocyte elastase inhibitor	SERPINB1	P30740
Matrisome-associated	ECM-affiliated Proteins	Annexin A5	ANXA5	P08758
Matrisome-associated	ECM-affiliated Proteins	Annexin A1	ANXA1	P04083
Matrisome-associated	ECM-affiliated Proteins	Annexin A11	ANXA11	P50995
Matrisome-associated	ECM-affiliated Proteins	Annexin A2	ANXA2	P07355
Matrisome-associated	ECM-affiliated Proteins	Annexin	ANXA3	D6RA82
Matrisome-associated	ECM-affiliated Proteins	Annexin A4	ANXA4	P09525
Matrisome-associated	ECM-affiliated Proteins	Annexin A6	ANXA6	P08133
Matrisome-associated	ECM-affiliated Proteins	Annexin A7	ANXA7	P20073
Matrisome-associated	ECM-affiliated Proteins	Complement C1q subcomponent subunit B	C1QB	A0A0A0MSV6
Matrisome-associated	ECM-affiliated Proteins	Complement C1q subcomponent subunit C	C1QC	P02747
Matrisome-associated	ECM-affiliated Proteins	Chondroitin sulfate proteoglycan 4	CSPG4	Q6UVK1
Matrisome-associated	ECM-affiliated Proteins	Hemopexin	HPX	P02790
Matrisome-associated	ECM-affiliated Proteins	Galectin-1	LGALS1	P09382
Matrisome-associated	ECM-affiliated Proteins	Galectin-3	LGALS3	P17931
Matrisome-associated	ECM-affiliated Proteins	Galectin-7	LGALS7	P47929
Matrisome-associated	ECM-affiliated Proteins	Mucin-2	MUC2	A0A0G2JR65
Matrisome-associated	ECM-affiliated Proteins	Mucin-5AC	MUC5AC	P98088

Matrisome-associated	ECM-affiliated Proteins	Mucin-5B	MUC5B	Q9HC84
Matrisome-associated	Secreted Factors	Angiopoietin-related protein 6	ANGPTL6	K7EKF6
Matrisome-associated	Secreted Factors	Cornulin	CRNN	Q9UBG3
Matrisome-associated	Secreted Factors	Fibroblast growth factor 1	FGF1	P05230
Matrisome-associated	Secreted Factors	Filaggrin	FLG	P20930
Matrisome-associated	Secreted Factors	Filaggrin-2	FLG2	Q5D862
Matrisome-associated	Secreted Factors	Hornerin	HRNR	Q86YZ3
Matrisome-associated	Secreted Factors	Protein S100-A14	S100A14	Q9HCY8
Matrisome-associated	Secreted Factors	Protein S100-A4	S100A4	P26447
Matrisome-associated	Secreted Factors	Protein S100-A6	S100A6	P06703
Matrisome-associated	Secreted Factors	Protein S100-A7	S100A7	P31151
Matrisome-associated	Secreted Factors	Protein S100-A8	S100A8	P05109
Matrisome-associated	Secreted Factors	Protein S100-A9	S100A9	P06702

From the 1335 detected proteins of the study, we used the Matrisome Annotator tool of the Matrisome Project database (http://matrisome.org/) to select proteins found in the human extracellular matrix. Of note, a couple of duplicate is present, COL6A3 and FBLN1, corresponding to proteins detected by 2 UniProt entries.

Supplemental Table S6. Abundance modifications of selected extracellular matrix and podocyte-specific proteins in transplant glomerulopathy

Protein name	Transplant glomerulopathy	Adjusted p-value	Glomerulitis	Adjusted p-value
**ECM proteins**				
NID1	1.12	0.30	0.95	0.85
COL4A1	1.35	0.32	0.83	0.69
COL4A4	1.10	0.97	0.92	0.53
LAMC1	1.24	0.28	0.89	0.95
LAMA5	1.12	0.35	0.82	0.69
LAMB2	1.15	0.30	0.96	0.87
TINAGL1	**1.97**	**0.03**	0.86	0.87
**Podocyte-specific proteins**				
NPHS1	**0.64**	**0.01**	0.76	0.40
NPHS2	0.62	0.40	0.92	0.97
PTPRO	0.73	0.29	0.82	0.30
PODXL	**0.57**	**0.01**	**0.69**	**0.01**
KIRREL1	0.70	0.09	1.08	0.69

The selection of the proteins is based on a previous proteomics study of ABMR [4], where a decrease in the displayed ECM proteins was seen in active ABMR compared to acute cellular rejection and acute tubular necrosis, with also a decrease of podocyte-specific proteins NPHS1 and PTPRO. From our dataset, non-parametric Mann-Whitney tests were performed to compare the protein expressions between caABMR and SG cases, reflecting changes occurring during transplant glomerulopathy. P-values were secondarily adjusted according to the Benjamini-Hochberg correction. A similar process was applied for glomerulitis with the aABMR/SG comparison. Abbreviations: aABMR, active antibody-mediated rejection; caABMR, chronic active antibody-mediated rejection; SG, stable graft control; ECM: extracellular matrix.

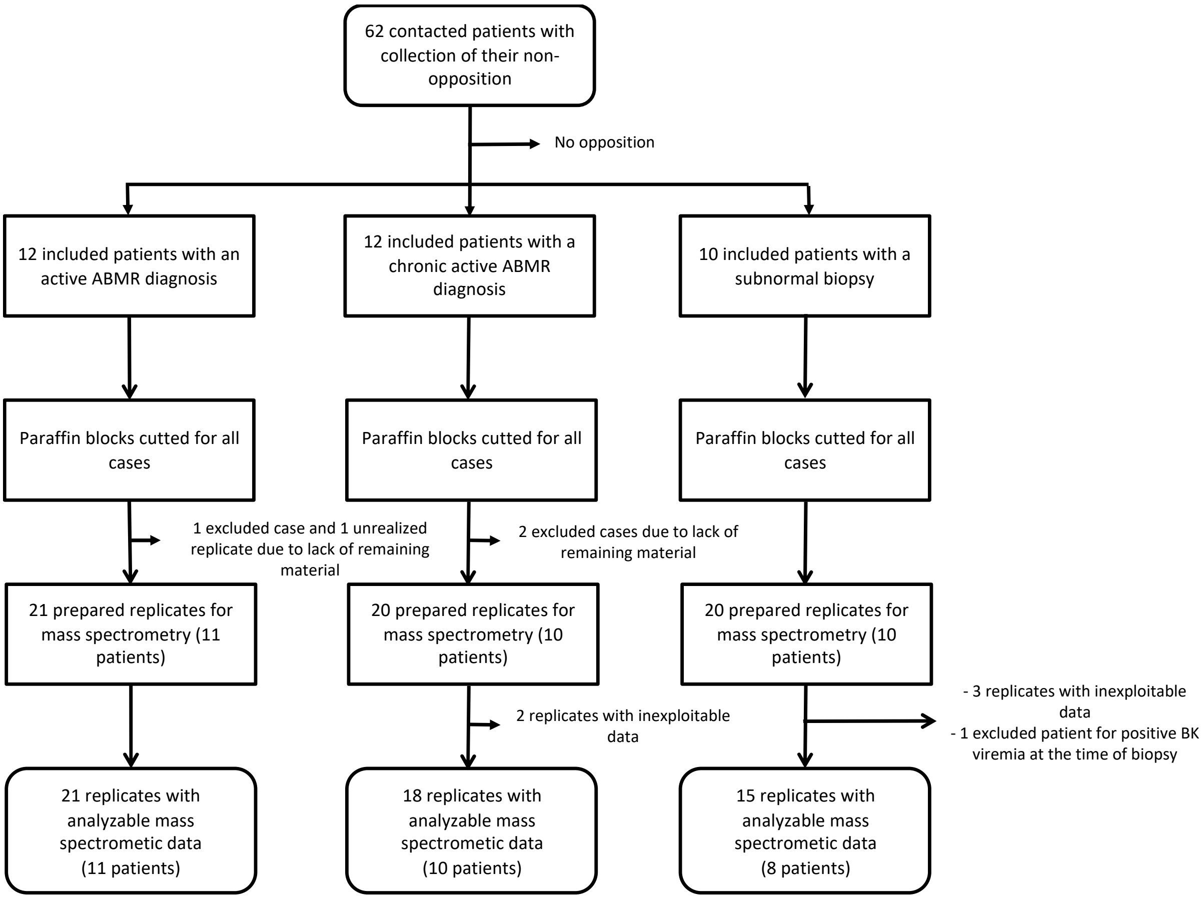

Supplemental Figure S1. Flow-chart of the study. Active antibody-mediated rejection (aABMR) cases were consistent with the Banff 2017 classification, but also all cases included in this group had anti-HLA donor-specific antibodies at the time of diagnosis and had a positive glomerulitis score (g>0). The inexploitable spectrometric data were due to overpressures in the pre-column of chromatography for some samples during processing, of unknown cause, which led to a misalignment of the spectrometric profiles and/or to poor protein identification performances.

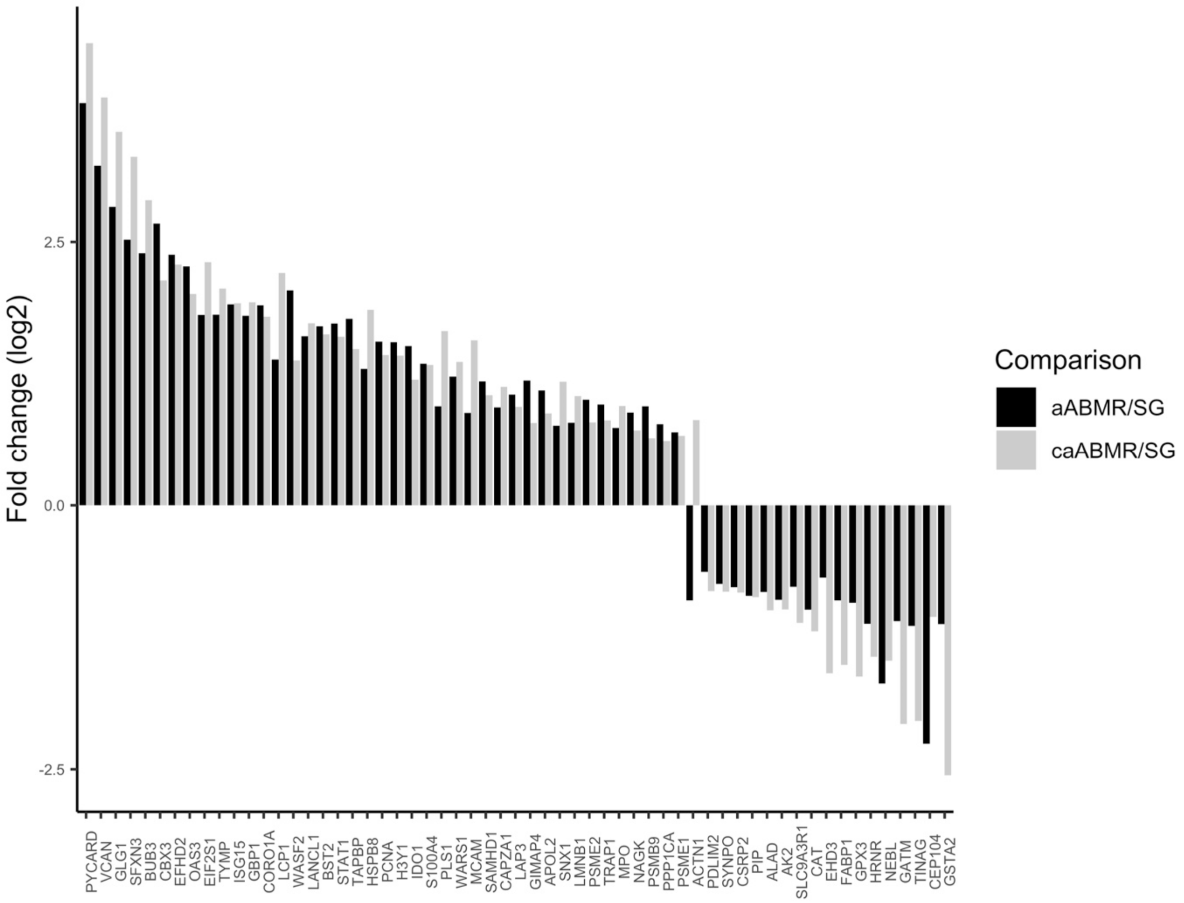

Supplemental Figure S2. Fold changes of the 59 proteins differentially represented in both antibody-mediated rejection groups compared to the stable grafts. The distribution is highly similar in the two comparisons, with no statistical difference between them (p=0.58, Wilcoxon signed-rank test). Differences were not normally distributed. Abbreviations: aABMR, active antibody-mediated rejection; caABMR, chronic active antibody-mediated rejection; SG, stable graft control.

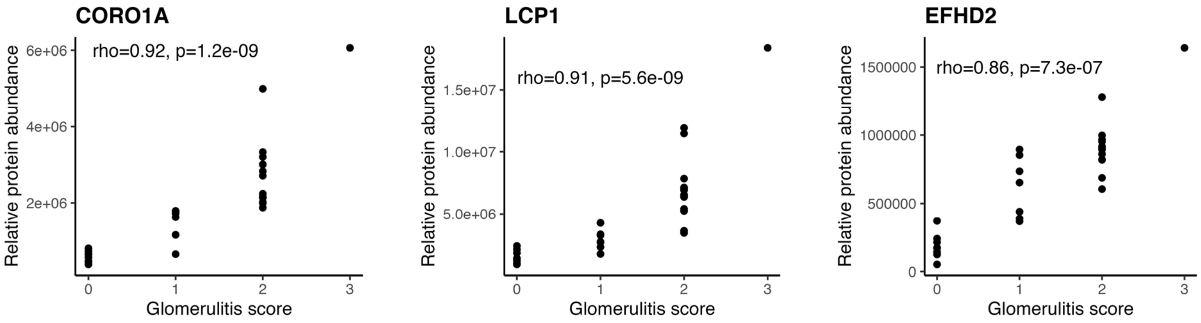

Supplemental Figure S3. Dot plots showing protein abundances by mass spectrometry depending on the glomerulitis score. Spearman's correlations were calculated, and the top-3 proteins of each correlation analysis are presented. P-values are adjusted according to the Benjamini-Hochberg correction. Glomerulitis is graded from 0 to 3 according to the Banff scheme.

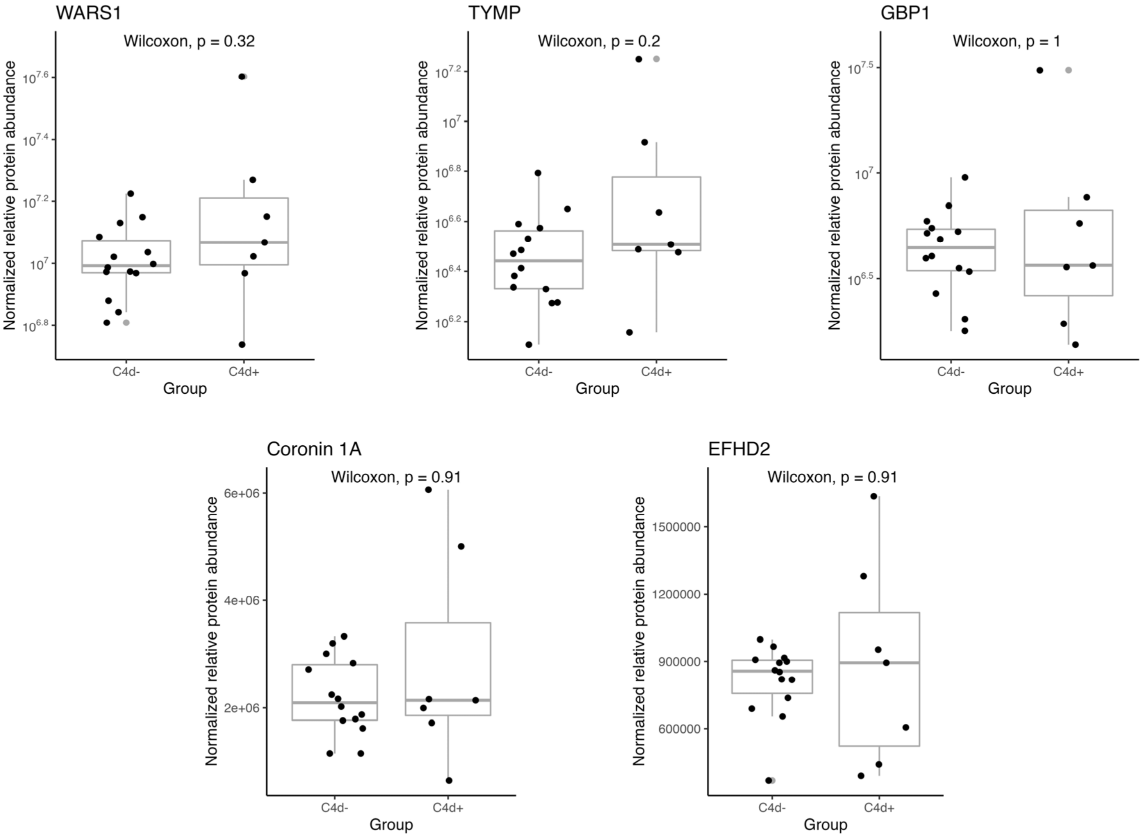

Supplemental Figure S4. Box plots showing protein abundances by mass spectrometry depending on the C4d status for the 5 proteins tested by immunohistochemistry. No statistically significant difference was seen for each antibody regarding the C4d status.

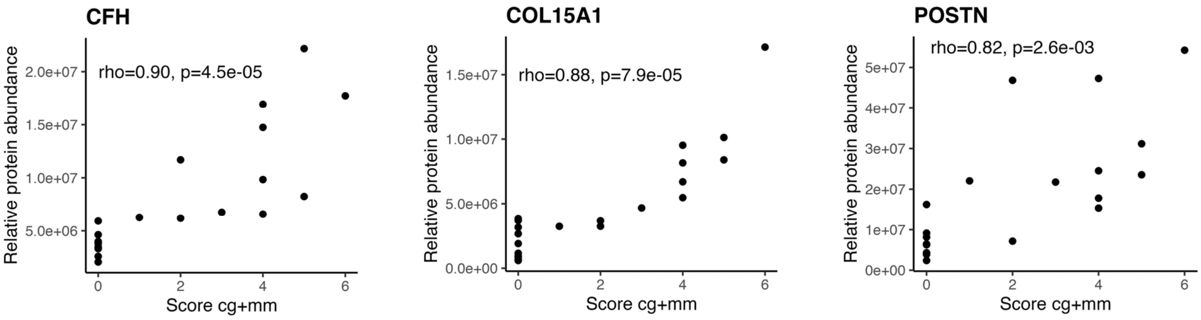

Supplemental Figure S5. Dot plots showing protein abundances by mass spectrometry depending on the cg+mm score, reflecting changes seen in transplant glomerulopathy. Spearman's correlations were calculated, and the top-3 proteins of each correlation analysis are presented. P-values are adjusted according to the Benjamini-Hochberg correction. Each individual score is graded from 0 to 3 according to the Banff scheme.
